# Switch Region for Pathogenic Structural Change in Conformational Disease and Its Prediction

**DOI:** 10.1371/journal.pone.0008441

**Published:** 2010-01-11

**Authors:** Xin Liu, Ya-Pu Zhao

**Affiliations:** The State Key Laboratory of Nonlinear Mechanics, Institute of Mechanics, Chinese Academy of Sciences, Beijing, China; Center for Genomic Regulation, Spain

## Abstract

Many diseases are believed to be related to abnormal protein folding. In the first step of such pathogenic structural changes, misfolding occurs in regions important for the stability of the native structure. This destabilizes the normal protein conformation, while exposing the previously hidden aggregation-prone regions, leading to subsequent errors in the folding pathway. Sites involved in this first stage can be deemed switch regions of the protein, and can represent perfect binding targets for drugs to block the abnormal folding pathway and prevent pathogenic conformational changes. In this study, a prediction algorithm for the switch regions responsible for the start of pathogenic structural changes is introduced. With an accuracy of 94%, this algorithm can successfully find short segments covering sites significant in triggering conformational diseases (CDs) and is the first that can predict switch regions for various CDs. To illustrate its effectiveness in dealing with urgent public health problems, the reason of the increased pathogenicity of H5N1 influenza virus is analyzed; the mechanisms of the pandemic swine-origin 2009 A(H1N1) influenza virus in overcoming species barriers and in infecting large number of potential patients are also suggested. It is shown that the algorithm is a potential tool useful in the study of the pathology of CDs because: (1) it can identify the origin of pathogenic structural conversion with high sensitivity and specificity, and (2) it provides an ideal target for clinical treatment.

## Introduction

**Figure 1:**
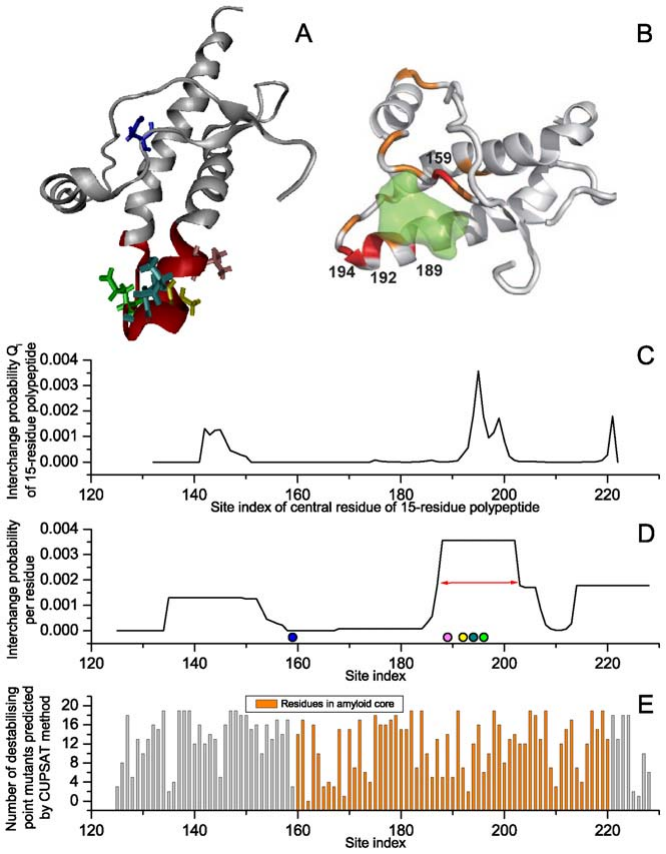
Results for human prion (PDB ID 1qm2_A, 104 residues in length). (A) Structure of prion in cartoon form. It has been reported that sites in bonds (159 blue, 189 magenta, 192 yellow, 194 olive, and 196 green) are important in hampering pathogenic changes in the prion. (B) Binding pocket for anti-prion compound GN8, overlaid in green [11]. (C) Interchange probability for each 15-residue segment indexed by its central residue. In our analysis, each residue is covered by at most 15 successive segments. To evaluate the significance of each residue site, we scored the interchange probability per site using the maximum interchange probability for the corresponding 15 polypeptides. The interchange probability for each residue site is shown in D. In A and D, the switch regions predicted are shown in red. (E) Significance per site for the stability of the prion predicted in the absence of evolutional information. Every type of point mutation is presumed for each residue in the prion protein, that is, 104
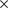
19 variants in total. The overall stability of each type of mutant was predicted using the CUPSAT algorithm [9]. The number of destabilizing mutants are reported per site. Residues in the core of the prion amyloid identified by site-directed spin labelling and EPR spectroscopy [13] are also shown.

Protein folding is a natural process wherein proteins undergo self-assembly through “folding” of their polypeptides into characteristic functional structures. At times, proteins fail to fold into their correct forms. When incorrectly folded proteins accumulate, they clump together; this is believed to be the cause of conformational diseases (CDs) such as Alzheimer's disease. Since the structure of a protein is the foundation of its biological function, such misfolding can either induce a disease-related error in performing the natural bio-function of an endogenous protein or produce a novel type of infection that is unfamiliar to the immune system. A well-known example for the former is the transmissible spongiform encephalopathy (TSE), a group of fatal neurodegenerative diseases caused by misfolding of the prion protein (PrP) [1], [2], [3]. The avian influenza virus [4] and A(H1N1) flu outbreak in April 2009 [5] are typical examples for the latter. Therefore, CDs are not rare, but are responsible for the development of wide range of diseases. Protein aggregation(i.e., amyloid deposition in tissues) is one of the typical features of many endogenous CDs. The aggregation-prone regions determine the tendency of proteins to aggregate and form amyloid fibrils [6], [7]. Such “hot spots” of fibril formation are usually abundant. As too many candidate target sites are involved, it is noisy and inappropriate for clinical treatment (e.g., in Figure 1E, half sites are in amyloid core of PrP). On the other hand, investigations conducted on such hot spots have mainly focused on the second stage of aggregation. The aggregation-prone regions tend to be blocked in the native state [8]. For an in-depth investigation on amyloid formation, the initial stage of the exposure of the hot spots, namely the unlocking mechanism in nosogenetic misfolding, should be the focus. The corresponding site is like a switch where the sensitive regions of the native structure begin to be exposed. Once identified, switch regions can be ideal targets for clinical treatment if they are not active sites. Moreover, for exogenous cases, such sites are also significant in identifying the pathology of a novel type of infection. Thus, there is a need to identify regions that are significant for the ‘disease-related’ stability of proteins.

In the last decade, protein stability has been of wide interest as a fairly mature subject. However, it still has much left to offer for the investigation of the nature of CDs. CDs are an evolutionary phenomenon that involve both protein misfolding and evolution. Prediction algorithms such as CUPSAT [9] can easily confirm that many sites are important for protein stability (accuracy 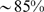, e.g. Figure 1E.). However, after hundreds of million years of evolution, many unstable protein mutants have been inhibited or forbidden by the selection pressure of evolution, and thus do not exist in the present CDs. Although unstable mutants can be predicted accurately, without the aid of evolutionary information, the importance of such variants may be estimated falsely, and too many candidate target sites would make clinical treatment difficult as well. Therefore, both evolutionary information and molecular stability must be considered in dealing with such an interdisciplinary subject.

Many proteins are involved in CDs. Some have been identified by clinical analysis, but there are also many unknown but suspected cases. As a prerequisite for in-depth research, clinical information directs the studies on CDs. While the clinical information is not sufficient because it requires sophisticated techniques and manipulations, it is difficult to be obtained even in countries with a high degree of medical expertise. Due to such restrictions, it was beyond the scope of the previous technical line to perform a systematic study of CDs. However, an accurate prediction of switch regions would cause advancement in systematic studies. For example, if a mutation occurs in a predicted switch region, then there is a considerable opportunity for a CD to develop. The scope of in-depth research could thus be usefully increased. Moreover, based on the predicted switch regions, we could evaluate the risk level of a candidate CD, determine the target sites for treatment, and then fix the protein in a stable conformation, overcoming the need for a complex and unnecessary analysis of the morbid conformation, and this would optimize clinical treatment.

**Figure 2:**
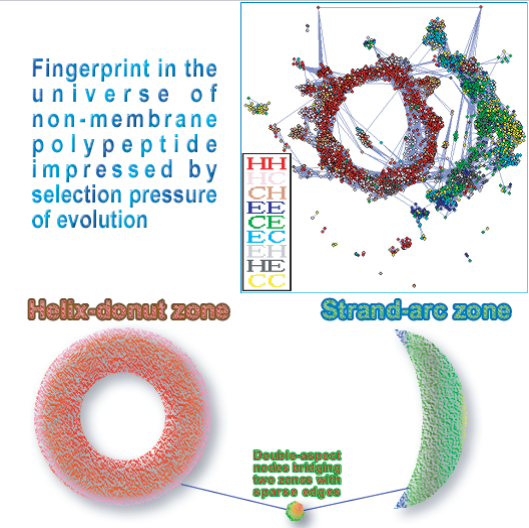
Sketch map of the topological features of homologous relationships for short residue segments in the whole universe of non-membrane proteins. The universe of non-membrane polypeptide is composed almost entirely of two nearly separated regions, a helix-donut zone and a strand-arc zone. The helix-donut zone consists of helix segments and N/C-terminal helix caps, while the latter is mainly comprised of 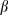-sheet segments and N/Cterminal strand caps. Two major regions are sparsely connected by bridge nodes that favor both types of folding, and can thus potentially cause conformational conversion. Insert: Donut-shaped fingerprint formed by vital nodes/polypeptides of the graph of polypeptide relationships (GPR) [10].

In a previous study, we have treated proteins as successive overlapping short residue segments, and have presented a graph of polypeptide relationship (GPR) [10]. The aim of that study was to identify detailed topological features of homologous relationships for short residue segments in the whole universe of non-membrane proteins. To construct a nonredundant graph, a data set composed of proteins that exhibit low sequence identity of less than 25% was analyzed. Short polypeptides were treated as nodes of a network. Remote homologous polypeptides were grouped together and connected by edges(see Appendix S1, About the graph of polypeptide relationship, GPR). As shown in Figure 2, the selection pressure of evolution has impressed distinct fingerprints in the universe of polypeptides, with the following features:

1. The polypeptide phase space is composed almost entirely of two nearly separated regions, a helix-donut zone and a strand-arc zone. The helix-donut zone consists of helix segments and N/C-terminal helix caps, while the latter is mainly comprised of 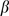-sheet segments and N/C-terminal strand caps.
2. The two parts are connected flimsily by sparse edges. Donors of these edges bridge the two zones.

As bridge nodes connect the two major regions, there should be a certain probability for these segments to be in one state, and a considerable probability to be in another state. In another word, they have a double aspect, that is, a potential to cause conformational conversion. It means that both requirements are satisfied by the knowledge system because there are distinct structural differences between a helix and a strand, and the evolutionary information on remote homologues has been considered for the construction of GPR. It is rational to investigate CD based on knowledge of the GPR.

In the present study, we used topological information from the GPR to predict switch regions responsible for the pathogenic structural changes. The algorithm was applied to most classical proteins responsible for diverse CDs. The results show that the algorithm is efficient and robust, and the predictions are consistent with clinical reports. To the best of our knowledge, this is the first accurate algorithm that can predict a number of CDs. Moreover, we analyzed protein NS1 of the H5N1 influenza virus, and found that local structural change is one mechanism responsible for the high pathogenicity and virulence of the H5N1 influenza virus. As a response to a recent outbreak, we also provided both theoretical and experimental evidence to illustrate the mechanism of the A(H1N1)/2009 influenza virus in overcoming species barrier as a result of a local structural change in its nucleoprotein. Moreover, the absence of immunological response to the switch region is responsible for the large number of potential patients and a high ratio of young sufferers.

## Results

We treated protein as successive overlapping short residue segments. For each residue segment, we evaluated its ability to develop pathogenic structural change by its probability to jump from the native helix-donut/strand-arc state to the other state. Segments with a high interchange probability are predicted to be switch regions. As illustrated in Methods, the algorithm is an inborn cross-validation for any query protein. A protein is predicted based on knowledge independent of itself.

Conformational change is a general disease related phenomenon. Some cases are classified as classical CD proteins due to the notable structural changes. There are also numerous disease-related proteins that are not classified as CD proteins but can be investigated in the context of CDs. It is difficult to identify proteins that are not associated with CD. Thus we tested the algorithm with the well-studied classical CD proteins and identified regions that cover the sites significant for initial pathogenic structural changes.

Since it is based on the knowledge of non-membrane proteins, the algorithm was designed for proteins in body fluids(PBF, proteins in both extracellular and intracellular fluids are involved.). The majority of the 31 disease-related proteins listed in refs [3], [14], [15], [16], [17] belong to this protein type. Among them, seventeen PBFs have usable protein structures, can be treated with the algorithm(see Appendix S1, About testing set). As an example, we report the detailed results for the prion. Results for the 16 other proteins are reported in the Appendix S1.

In 2007, Kuwata et al. [11] reported that the intercalation of an anti-prion compound GN8 to the region N159-V189-T192-K194-E196 could inhibit the pathogenic change process of the prion. The five sites should be significant in triggering conformational conversion, that is, they should act as switches for the PrP. We evaluated the interchange probability for each 15-residue polypeptide of human PrP. As shown in Figure 1C, a peak for the interchange probability occurs in the window with central site 195. Thus, sites 188–202 should be responsible for the origin of the conformational changes. Four out of the five aforementioned switch sites are within this region. Moreover, as shown in Figures 1A and 1B, compared with the functional area reported by Kuwata, we found that the predicted switch region corresponded exactly to the main body of the binding pocket for GN8, which means our prediction is quite accurate.

Switch region is a concept based on clinical cases that come forth under the selection pressure of evolution and that actually occur in life. It is not appropriate to define switch regions using information on artificial unstable mutants, in which evolutionary pressure is neglected. Therefore, data from experiments on point mutations were not considered in identifying switch regions. Different from fibril-forming hot spots that are usually identified by observing the core of amyloid fibrils, we identified initial sites of conformational changes through reports in which a switch role is evident, such as experiments to inhibit the pathogenic change process (prion) and disease-related point mutations observed in clinical practice. For some proteins, there are many residue sites where disease-related mutations can be clinically observed, that is, where disease is induced. If a region is significant for the initiation of pathogenic structural conversion, the density of this disease-related site should be high. Consequently, it is rational to define a switch region as a segment with the highest density of disease-related sites. In this way, we were able to identify switch regions for low-density lipoprotein, cystic fibrosis transmembrane conductance regulator, apolipoprotein AI, fibrillin-1, superoxide dismutase, crystallins, and hemoglobin (see reference in Table 1). There are too many such sites for hemoglobin, and the differences in density are not obvious. Thus, only sites corresponding to highly unstable disease-related mutants were used in the identification for hemoglobin. For the other proteins, the identification was easier and less controversial. There is ample credible proof in the literature that is widely accepted for each switch region we have defined. The diversity in identifying switch regions exhibits the variety in biology.

**Table 1:** Switch regions predicted for pathogenic structural changes of different disease-related proteins.

Protein	Disease	PDB ID chain	Predicted region	Prediction accuracy and proof [reference]
**Proteins in body fluid**				
Insulin	Injection-localized amyloidosis	1AI0_A	6–20 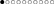 IP = 0.0019	**Accurate.** Native disulfide bonds contributed by the cysteines in region 6–20 are retained in fibril, providing substantial constraints to refolding. The corresponding segment plays a significant role in forming initial aggregates of insulin amyloid, i.e. a switch region [29]
Prion	Creutzfeldt-Jakob & kuru disease in humans	1QM2_A	188–202 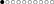 IP = 0.0036	**Accurate.** Intercalation of anti-prion compound GN8 to region N159-V189-T192-K194-E196 can hamper the pathogenic structural conversion process [11]. This indicates that sites 189, 192, 194 and 196 are the kernel of the switch region for prion.
Apolipopro-tein AI	Familial amy-loid polyneuro- pathy, visceral amyloid	2A01_A	1–15 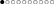 IP = 0.046	**Correct.** Helices A:1-43 govern the structural stability of lipid-free Apo-AI [30]. In these helices, some point mutations at sites 3,10,13, and 26 result in various clinical consequences [31]. Among these sites, three of four are in the region we predict.
Calcitonin Ct	Medullary carcinoma of the thyroid	1BYV_A	16–31 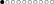 IP = 0.058	**Correct.** Segment 15–21, especially residues 18 & 19 governs fibril formation and the biochemical properties of human calcitonin [32], [33].Joint mutations Y12L-N17H-A26N-I27T-A31T [34] hamper pathogenic refolding, resulting in non-amyloidogenic analogues of human Ct.
Cystatin C	Hereditary cerebral angiopathy	1G96_A	61–74 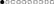 IP = 0.073	**Correct.** According to clinical reports, the most important mutation is at 68 Leu 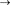 Gln, which is associated with a severe conformational disease and causes massive amyloidosis, cerebral hemorrhage and death in young adults [35], [36]
Hemoglobin	Severe hemolytic anemia	1XZ2_B	99–125 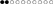 IP = 0.14	**Correct.** In all point mutations related to hemolytic anemia there are four highly unstable variants (V60E, L110P, A115D, Q127R). Three of these are in or close to the region we predict. This means that the predicted region is significant for the stability of the hemoglobin 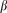 -chain [37].
Gelsolin	Finnish hereditary systemic amyloidosis	1RGI_G	243–264 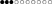 IP = 0.21	**Accurate.** Amyloidogenesis of plasma gelsolin is triggered by metalloendoprotease cleavage. In aberrant endoproteolytic release of amyloidogenic fragments, the cleavage site is either A 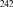 –M 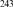 or M 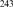 –L 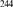 [38].
Lysozyme	Familial visceral amyloidosis	1W08_A	41–57 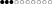 IP = 0.22	**Correct.** According to [39], structural rearrangement in region 45–51 is small in ordinary mutants, but very strong in disease-related variants. Consequently, sites 45–51 should be a switch region for lysozyme. These sites are within the region we predict.
Fibrillin-1	Marfan syndrome	1EMN_A	2146–2164 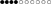 IP = 0.33	**Correct.** In the motif, there are three sites(D2127, N2144, C2151) for which marfan syndrome related point mutations have been reported [40]. Two of them coincides with our prediction.
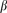 micro-globulin	Hemodialysis amyloidosis	2VB5_A	7–23 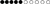 IP = 0.47	**Correct.** Connected to the predicted region, 21–31 is the amyloido- genic core fragment of 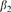 microglobulin [41], [42], [43]. Acidification, e.g. at site 17(N 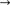 D ), is necessary for amyloid fibril formation from both wild-type 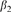 microglonulin and its variants [44].
Superoxide dismutase, SOD	Amyotrophic lateral sclerosis	2C9V_A	30–46 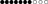 IP = 0.66	**Correct.** There are a total of 26 residue sites for which disease-related point mutations have been reported [45]. Segment 37–48 is the region with the highest density of these 26 residue sites. The ratio of site density between this and the next top region is 3∶2.
Transthyretin TTR	Familial amyloid neuropathy	1DVQ_A	46–69 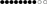 IP = 0.72	**Correct.** L55P is the most notorious mutant, with onset of clinical disease at about 20 years of age [46].In comparison, the age of onset is about 30 for V30M carriers and 80 for wild-type subjects. More- over, site 55 is important in the pathway for TTR polymerization to amyloid fibrils [47].
p53 tumor suppressor protein	Various cancers	2FEJ_A	191–205 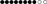 IP = 0.78	**Qualitatively correct.** Most cancer mutations are located in the DNA-binding core. Thus, the N-terminal half (residues 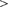 195) is important in conserving p 53 activity [48], [49]. In agreement, we predict higher interchange probability for the N-terminus. While highly destabilizing mutants are interspersed along the sequence. There should be several switch regions in p53 [50]. Our basic one-switch hypothesis cannot cope with such extremely complicated case.
Serpins	Antithrombin deficiency thromboem- bolic disease	1E04_A	372–386 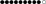 IP = 0.87	**Correct.** The predicted region is on the N-terminus of the reactive loop that is vital for the biological properties of serpins, e.g. 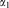 –antitrypsin. Site 381 plays an important role in stabilizing native, inserted, and activated states of serpins [51].
Crystallins	Cataracts	1HK0_X	14–36 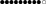 IP = 0.88	**Correct.** There are five residue sites for which mutants associated with congenital cataracts have been reported, three of which are in the region we predict. Substitution P23T is reported as the cause of pivotal local conformational and dynamic differences [52]
**Domain of membrane protein extends out into body fluid**				
Low-density lipoprotein receptor	Familial hyper-cholesterolemia, premature heart disease	1AJJ_A	25–39 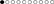 IP = 0.0094	**Correct.** There are eight residue sites for which disease–related point mutations have been observed. Six of them are in the region we predict [53]. This is thus the most important region for initiation of pathogenic structural conversion.
Cystic fibrosis transmembrane conductance regulator CFTR	Cystic fibrosis	1XMI_A	546–561 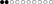 IP = 0.11	**Correct.** According to the cystic fibrosis mutation database [54], segment 542–560 is the region with the highest density of missense mutations responsible for cystic fibrosis. The ratio of site density between this and the next top region is 2∶1.
**Membrane proteins**				
Amyloid- 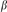 precursor protein APP	Alzheimer's disease	1Z0Q_A	699–713 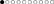 IP = 0.0026	**Qualitatively correct.** Most pathogenic residue mutations occur in region 692–724, where the top two high density zones are 713–717 and 692–694 [55]. The predicted region coincides with such clinical reports. Since the structural data end up at A713, and all these regions embed in membrane [56],the prediction is not quite accurate.
**Membrane-associated proteins**				
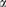 -synuclein	Parkinson's disease PD	1XQ8_A	89–124 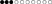 IP = 0.25	**Maybe correct.** Three missense mutations(A30P, A53T and E46K) are associated with familial PD [57]. While most PD patients carry wild-type 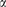 -synuclein.Therefore PD might be triggered at other sites. As a hotspot of recent research,the 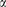 -synuclein-membrane interaction was found to affect bilayer structure,stability, and fibril formation [58]. Both N- and C-terminals of 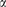 -synuclein are tightly associated with membrane [59], important. #89-124 belongs to the C-terminal region.
Branch-chain 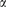 -ketoacid dehydrogenase complex BCKD	Marple syrup urine disease MSUD	1U5B_A	146–160 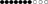 IP = 0.68	**Wrong.** Branched-chain keto acid dehydrogenase is a multienzyme complex associated with the inner membrane of mitochondria.There are five disease-related point mutations [60]. None of them belongs to the region predicted.
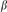 -hexosaminidase	Tay-Sachs disease	2GJX_A	95–109 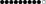 IP = 0.87	**Wrong.** 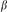 -hexosaminidase is one part of a complex that degrades the lipid GM2 ganglioside.The malfunction of its 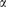 -subunit brings the ac-cumulation of GM2 ganglioside in lysosomes, then a neurons damage. None of 51 pathogenic point mutations [61] is in the region predicted.

Interchange probabilities(IP) for the switch regions predicted are also listed, with sketch maps increasing 0.1 per ‘
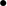
’.

We predicted the potential switch region in CDs proteins and found that they could map to actual switches very well. For low-density lipoprotein and cystic fibrosis transmembrane conductance regulator, the corresponding motifs extend out into the body fluids, aiming at the molecules therein. These modules work far from the membrane, largely in a non-membrane-like environment, therefore they can also be treated by the algorithm. As shown in part one of Table 1, for proteins in body fluid or motifs extending out into the body fluids, there is evidence of the key contributions in initiating pathogenic structural changes for most of the predicted switch regions. The prediction results and clinical reports are in very good agreement. Aside from the neutral prediction for the extremely complicated tumor suppressor p53, 16 out of the 17 proteins are correctly predicted, with a success rate (sensitivity and specificity) of approximately 94%. There are a total of 2649 residues in the 17 proteins, in which a small proportion (302 residues, 11.4%) was predicted as residues in the switch regions. Therefore, the algorithm is highly sensitive and specific for the region of switch/true signal.

To clarify the available range of this algorithm, we show the results of the proteins that fit poorly in the second part of Table 1, wherein the limitation is shown by the predictions especially in branch-chain 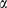-ketoacid dehydrogenase complex and 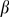-hexosaminidase. These proteins either interact tightly with the membrane or aim at the molecules on the membrane, namely they have significant interactions with the membrane environment. Therefore, the knowledge system of the present algorithm is unsuitable, resulting in unsatisfactory predictions.

The purpose of the algorithm is to identify the riskiest region of a protein, and to construct a foundation for further investigation. Although per residue prediction is not in the scope of this study, we found that the sites tightly related to CD are abundant in the region we predicted. In the test set of the 17 proteins, 85 residues are tightly associated with CDs. About 52.9% (45) of them are in the region predicted. In the 302 residues involved in the switch regions predicted, 14.9% of them have been proven to be tightly disease associated. Whereas, for those not in the switch regions predicted, only 1.7% of the residues are tightly associated with CDs.

Interchange probability is a numerical measure of the incidence of a disease in terms of knowledge of protein evolution. As shown in the first part of Table 1, cases with low interchange probabilities in the switch regions are highly fatal (expect injection-localized amyloidosis, which does not exist in nature and is induced artificially). For cases involving prions, cystatin C, and hemoglobin, patients who do not undergo clinical treatment usually die at a very young age, without having the opportunity to transmit their genes to their offspring. These endogenous proteins are abundantly expressed in vital tissues. Because of the high risk, they must remain very stable. Protein evolution ensures such stability through low interchange probability. However, once misfolding occurs, the result can be fatal. This may be one of the mechanisms by which nature controls life through evolution.

## Discussion

We have presented an algorithm for predicting switch sites in pathogenic structural changes in CD. As our algorithm is not sensitive to individual variants, the corresponding therapeutic regimes should be suitable for most patients. Moreover, as it approximately characterizes the incidence of a disease regarding evolution, interchange probability can surely be used to identify proteins related to CD. This will be published elsewhere.

The success of this study is due to the high accuracy in identifying remote homologous relationships. It ensures correctness in calculating interchange probability. In 2008, we found that, in a protein family, the intramolecular hydrophobic force network of each member/protein has some common, family representative features by which a model of family representative hydrophobic force network can be constructed. According to this model, new remote members (with sequence identity no more than 30%) of a protein family can be designed computationally, together with family representative biochemical properties. At least 80% of the remote members designed have similar bio-activities with wild-type proteins (to be published elsewhere). According to such computational designing and experimentation, it was shown that this model/theory has caught some evidence for protein evolution [12]. The present work adopts this model, gaining great improvement in the quality of remote homologue identification [10], [18].

**Figure 3:**
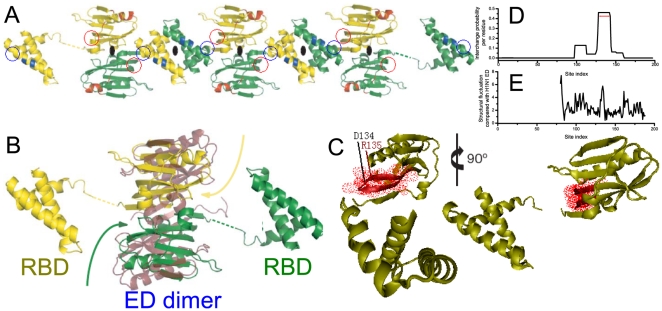
The formation of H5N1 NS1 chain induced by local conformational conversion of residues around ED:R135. (A) NS1 chain formed by the units shown in B [4]. In each unit, the ED of NS1 molecule interacts with each other, forming an ED dimer. The RBDs of each unit interact with the RBDs and ED dimers of the neighbouring units. (B) Superposition of H5N1 NS1 dimer with the H1N1 NS1 ED dimer(in ruby; PDBID, 2GX9), demonstrating the twisting motion (curved arrows) of the H5N1 ED monomer, with respct to H1N1 ED. (C) Illustration of the predicted switch region for H5N1 NS1(PDBID, 3F5T), shown in red and dots with central site R135. (D) The interchange probability for each residue site of full-length H5N1 NS1 molecule. (E) A comparison of the crystallographic C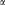 atom deviations(Å) for H5N1 NS1 ED and H1N1 NS1 ED. There is notable structural rearrangement around residue D134. Although a large structural fluctuation occurs at the N-terminal of H5N1 NS1 ED, which is also part of the interface between RBD and ED dimer, it should not be responsible for the formation of NS1 chain because nearly identical structural rearrangement also exists in the NS1 of low pathogenicity avian influenza virus H12N5(A/Duck/Albany/76; PDBID, 3D6R; The C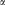 atom deviations between H12N5:3D6R and H5N1:3F5T are at most 3.5 Å for these N-terminal residues), but induces no high pathogenicity. In A, the binding sites of RBD induced by conformational conversion of ED are marked with red circles.

The high success ratio makes the present algorithm a powerful tool for use in cases that lack clinical reports. As an example, we present several clues for the study of the highly pathogenic H5N1 influenza virus. The non-structural protein NS1 was suggested to be associated with the increased pathogenicity and virulence of these strains. NS1 consists of two domains, a helix-rich double-stranded RNA binding domain (RBD) and the 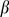-sheet-rich effector domain (ED), separated through a linker. It is an antagonist of the antiviral type-I interferon induction in the host. Sixty percent of human deaths in an outbreak in Vietnam were related to the NS1 from an H5N1 strain (A/Vietnam/1203/2004). It was claimed that, instead of distinct dimeric units formed by ordinary NS1, H5N1 NS1 forms a molecule chain (Figure 3A), which assembles into three-chain tunnel consequently, resulting in a fatal mechanism that resists antiviral interferon response [4]. This is due to a significant large-scale structural alteration of the ED (Figure 3B). For the key mechanism of the formation of the NS1 chain, several possibilities have been proposed focusing on the dimeric interface of the H5N1 NS1 ED, the formation of the RBD dimer, and the length of the linker, respectively.

According to our analysis, the region most important for the structural change of H5N1 NS1 focused around the resides near R135(#128-143; Figure 3C, D). Compared with the structure of H1N1 NS1 ED (a low pathogenic case), this segment has the most striking structural rearrangement(Figure 3E). Such structural rearrangement results in the formation of a new RBD binding sites. Accordingly, NS1 chain with alternating RBD and ED dimers is formed when the RBD and ED of each NS1 molecule interact with their respective domains from nearby NS1 molecules (Figure 3A). This is induced by the structural rearrangement of the residues around H5N1 NS1 ED R135 that creates the binding sites. More significantly, the maximum interchange probability is high, which is 0.46 for H5N1 NS1 protein(Figure 3D). In an evolutionary aspect, there is a remarkable opportunity for other highly pathogenic variants to emerge due to the conformational conversion around R135, that is, inducing more health-related problems. We suggest this region to be further studied.

Since its emergence in April 2009, the A(H1N1) virus has raised significant global health concerns. Although many people in public health organizations have been mobilized around the world, the trend of this pandemic is much powerful. After about 7 months, over 503536 people were already infected with at least 6260 deaths(13 Nov 2009). The most dangerous feature of this “triple reassortant” virus (a hodgepodge of North American swine, avian, and Eurasian swine flu virus) is that the strain has overcome the species barrier, this makes humans a reservoir of the virus too. As it has spread throughout the world, billions of people are at risk.

The RNA segment 5 of the influenza A virus encodes nucleoprotein (NP), which binds to the viral RNA and can be packaged into ribonucleoproteins (RNPs). NPs, along with three polymerase proteins, PB1, PB2, and PA, play important roles in the viral replication life cycle. NPs also function as a key adapter molecule between virus and host cell processes, and is suggested to be decisive in host range restriction [19], [20], [21](see Appendix S1, Protein selection for 2009 A(H1N1)). Since several epitopes on NPs have been found to be associated with the escape from human cytotoxic T lymphocytes(CTLs), NPs are believed to prevent viral replication from the attack of the host immune system [22], [23].

The NP structure is an important feature that determines the reservoir of a virus [21], for example, in a region where T-cell recognizes. A conformational conversion means an altered/new target in immunology, which can result in an alteration of the host range potentially. Therefore, the overcoming of the species barrier is also a case of CD. The switch region of structural change should correspond to the sites significant in restricting host range. Based on the high-quality theoretical analysis given by the present algorithm, we attempt to uncover the mechanism of strain 2009 A(H1N1) in overcoming the species barrier, and discover the reason of its high host population.

**Figure 4:**
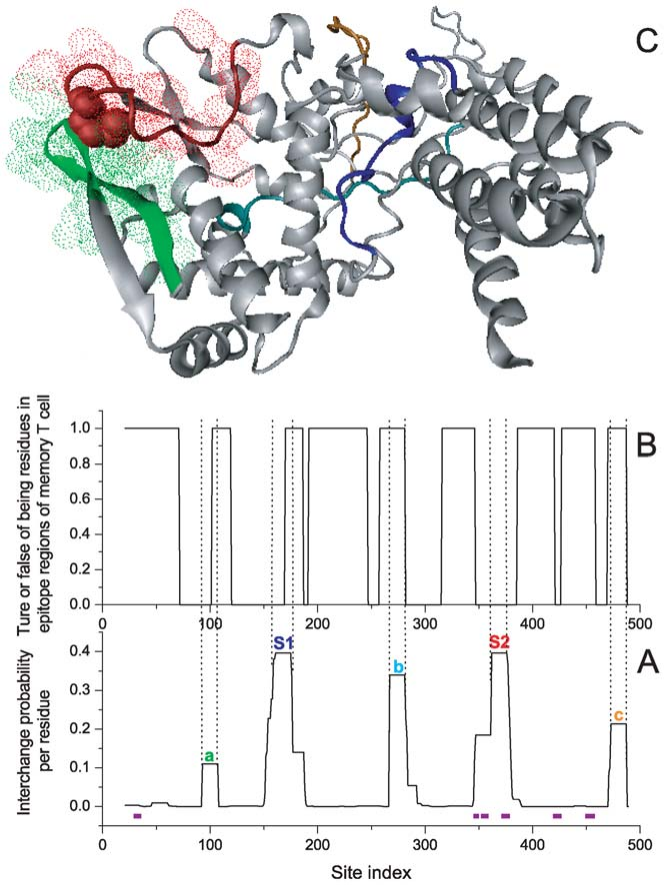
Contribution of NP in overcoming species barrier for the 2009 A(H1N1) virus. (A) The interchange probability for each residue site of NP(PDBID, 2IQH_A). S1 and S2 are double switch regions. Phylogenetically important regions are shown in purple. (B) Coverage of epitope regions for memory T cell immune responses. Residues involved in the epitope regions reported by ref. [26] are assigned value 1 (true), and 0 (false) otherwise. Except S2, all peak segments contain residues involved in epitope regions of healthy individuals (epitopes are observed from 90 persons in total). The epitope data are credible because they are obtained by systemic ex vivo experimential analysis of the influenza A virus-specific memory T cell responses (H5N1). Moreover, data from an independent research group coincide with them (H3N2, data not shown). (C) Structure of NP where segments are colored as those in A. As a marker, residue 371 at the C-terminal of S2 is shown in bulk. The C-terminal of S2 and the human host segment ‘a’ are tightly adhered.

According to Figure 4A, there are two switch regions in NP (S1[160–174], and S2[360–374]; analyzed by a homologue of the NP of A/California/04/2009(H1N1), the sequence identity is 83%, PDBID_Chain: 2IQH_A). We searched the NCBI database (http://www.ncbi.nlm.nih.gov, using blast tool [24]), and found that segment S1 of virus 2009 A(H1N1) has been expressed by various strains (including human host) for many years. The current outbreak might not be due to the invalidation of the human immune system against the segment. Therefore, S1 might not be responsible for the present host range alteration. On the contrary, segment S2 of virus 2009 A(H1N1) was only expressed by strains of avian and swine flu viruses, but never in the human strain before the current outbreak, that is, a novel target for the human immune system could potentially arouse host alteration.

Actually, for residues around the switch region S2, the experimental evidence for the key role of host restriction is quite prominent. In virology, the protein has some phylogenetically important regions (PIRs), which are believed to be important in host selection and are considered host-specific functional regions of the NP. As shown in Figure 4A, three out of six PIRs are around switch region S2, which is also the only region where multiple host types are related (human, swine, avian, and equine) [25]. Therefore, the map from the switch region of conformational conversion, with positions belonging to S2 in particular, to significant sites of host restriction is quite a success, although we still cannot illustrate the host type in the above analysis (Here, we only focused on segment position, but not the sequence details. See the following paragraph for such details.).

For the novel influenza virus, the human host type is caused by a special feature of segment S2. Because the segment belongs to the most significant region for host restriction, the host becomes very sensitive to residue details in S2. Therefore, the special segment TRGVQIASNENVETM of the 2009 A(H1N1) S2 is vital, that is, a detailed analysis of its human reservoir property is needed. Although the segment mainly exists in strains of avian and swine flu virus, it has the capability to be expressed in human strains. (The fact has been observed in the current outbreak.) As evidence, all single point mutations of this segment, the nearest homologues in the NCBI database have been expressed by different human strains (The details are shown in Table S1 in Appendix S1). Thus, this segment essentially belongs to the human strain, and the current alteration to the human host type is reasonable.

A potential mechanism of the 2009 A(H1N1) virus in overcoming the species barrier has been illustrated. Most viruses that express the segment TRGVQIASNENVETM belong to swine/avian strains. While the 2009 A(H1N1) virus is predisposed to human host, there should be certain factor that alter the preference and induce the virus to become human strain. As shown in Table S1 of Appendix S1, the human host mutations are on the C-terminal residues of S2, that is, a PIR [25]. According to the three-dimensional protein structure shown in Figure 4C, these residues adhere most tightly to segment ‘a’ GPIYRRVDGKWMREL 94–108, which is also prone to arouse conformational conversion and is mainly expressed by strains of the human host. The human dominant segment ‘a’ can induce S2 to be human specific, that is, the cooperation of structural conversions between ‘a’ and S2 is a potential inducement mechanism. As a coincidence, there is not a joint segment of ‘a’ and S2, GPIYRRVDGKWMREL-TRGVQIASNENVETM, in the NCBI database before the present outbreak. This implies that the human-specific alteration might be due to a new combination, which induces the structural conversion of S2 by adhering a human-dominant residue segment ‘a’.

Another dangerous feature of 2009 A(H1N1) is the lack of knowledge why so many people have been infected by the virus. Virus infection can induce the T cell-mediated immunity in the host, which targets the highly conserved internal proteins of the virus, such as M1, NP, and PB1 [26]. As a result, most healthy human adults possess immunological memory against the influenza virus due to the exposure to seasonal human influenza viruses. For example, a segment similar to the S2 of the 2009 A(H1N1) virus can induce the immunoreaction of memory T cell through epitopes such as “KLSTRGVQIASNEN” 357–370 (this epitope is taken from the A/Puerto Rico/8/34(H1N1) strain [27], has 11 residues identical to those of the S2 of 2009 A(H1N1) NP). As shown in Table S1 of Appendix S1, single-point mutants of S2 of the 2009 A(H1N1) virus have been expressed in some classical human strains. Most of the corresponding A(H1N1) strains, such as A/New Jersey/8/1976(H1N1), A/Brevig Mission/1/1918(H1N1), and A/Puerto Rico/8/34(H1N1), appeared at least 20 years ago. People who have been infected by these viruses may be immunocompetent against the present virus through epitopes related to S2. There is not such immunological memory for people who have never met such epitopes. Because viruses whose S2 is similar to that of 2009 A(H1N1) have been silent for a long time, many healthy individuals have no immunological memory against S2 due to the lack of exposure (as these have not globally prevailed for a long time). In many healthy adults(Figure 4B), there is no epitope corresponding to residues in the region around S2, such that there is no memory T cell-mediated response to segment S2 of the 2009 A(H1N1) NP. This may be the cause of the large host population and the tendency of infection in young people. Since correlating with several typical global pandemics (Table S1), segment S2 is extremely important for host range restriction, and is a common feature of pandemic flu.

## Methods

Our method is based on the assumption that the site responsible for the initial pathogenic structural change in a CD likely belongs to a segment related to the double-aspect nodes of the GPR. Namely, some of the edges emanating from a double-aspect node connect directly to the helix-donut zone, while others connect to the strand-arc zone. Based on this idea, for each segment of a query protein, we can find a set of its homologues in the nodes of the GPR. Since they have similar biological properties, the set of homologues can be deemed to be ‘copies’ of the query polypeptide in the universe of polypeptides characterized by the GPR. Information on the initiation site can be deduced from the double-aspect degree, which is evaluated from the connections of this set of nodes. The algorithm comprises three steps.

### 1. Identify Remote Homologues of the Query Polypeptide in the GPR

Our analysis is based on the GPR, from which a database 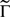 was constructed. Each node of the GPR acts as an entry heading, and is assigned an entry index. The second part of an entry contains indices of nodes that are directly connected to the node labelled as the entry heading by one edge in the GPR. Consequently, in database 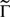, each node 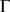 of the GPR was mapped to an entry in the form ‘ Index, 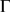; Index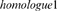, Index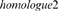, Index
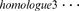
’.

A query protein is treated as successive residue segments. As the residue–residue correlation is notable in 15-residue segments [28], we used a window width of 15 for further consideration. By sliding a 15-residue window along the protein sequence, each segment of the query protein serves as a query polypeptide. Using the method described in Section 2.1 of reference [10], we searched for remote homologues (RHs) of the query polypeptide in set {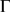}. Multiple features were investigated for this search algorithm, such as low level of sequence identity (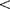
26.7%), high levels of similarities in sequence, three-dimensional structure, and surface residue distribution. Only segments with a credible relationship to a query polypeptide were collected in the final RH set. This led to a set of RHs 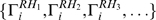 for each query polypeptide 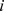, where a query polypeptide is indexed by its central residue 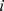. Each member of this set corresponds to an entry in the database 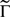. Then, a set of entries related to query polypeptide 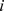 was obtained:
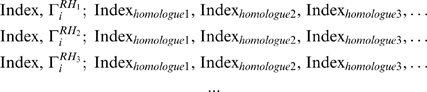
(1)


### 2. Evaluate the Opportunity for Interchange between the Helix-Donut and Strand-Arc Zones

For each query polypeptide, we evaluated its propensity for structural change using information for the entries obtained in the first step. For a 15-residue polypeptide, we can define subsegment 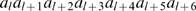 (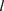 = 0 or 8) to be in state 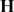 if more than three residues are in a helix conformation, in state 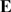 if more than three residues are in a strand conformation, and in state 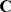 otherwise. Consequently, nine states are defined: 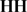, 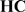, 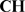, 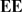, 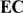, 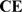, 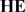, 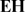, and 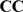. Figure 2 shows that nodes of 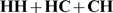 correspond to the main body of the helix-donut, and nodes of 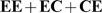 are largely in the strand-arc zone. Thus, we can deduce the position of a segment in the polypeptide phase space from its secondary structure. We assumed that samples in 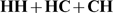 belong to the helix-donut zone, whereas those in 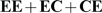 are in the strand-arc.

For a query polypeptide 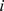, we evaluated the probability of belonging to the two zones by node indicated by the indices in the second parts of the entries in Eq. (1). Polypeptides indicated by these indices were collected as set 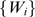 for each query polypeptide 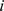. Using information on these correlative polypeptides, we can evaluate the probability of query polypeptide 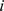 being in zone 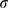 as: 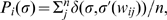 where 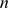 is the size of set 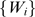, 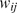 is a member of set 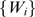 with index 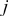, 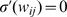 if 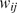 belongs to 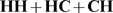 and 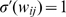 if 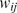 is in 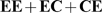, and 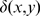 is a step function with 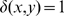 for 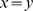 and 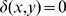 otherwise. 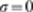 corresponds to the helix-donut zone, and 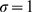 to the strand-arc zone.

In our viewpoint, every query polypeptide has the opportunity to be either in the helix-donut zone or in the strand-arc zone, or in the other zones. Information provided by the structure in hand is only related to one state of a query polypeptide. There is opportunity to jump to other states. Because the donut-arc part contains nearly entire nodes of GPR, an investigation of the interchange between the two zones will be representative and accurate enough. For query polypeptide 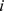, the probability of interchange between a helix-donut and a strand-arc zone can be evaluated as,
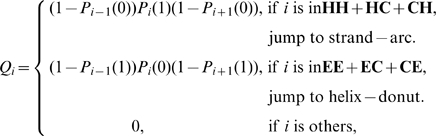
(2)where 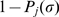 denotes the probability that segment 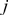 is not in state 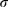. It is reasonable that a polypeptide with a higher interchange probability is more prone to drifting into the structural change related to a CD.

### 3. Filter of Conformational Changes Allowable under Normal Conditions

Due to thermal motion, a protein has a normally flexible structure that continually changes at room temperature. Helices and strands can extend or shrink to a moderate degree. Such structural conversion is vital for the biological functions of protein molecules. For a segment with both helix and 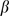-sheet conformations, the two types of secondary structure are interchangeable under normal conditions. Such facile extension/shrinking of the inborn secondary structure should not cause a disease; otherwise the species would have been lost during evolution [8]. Therefore, the corresponding sites are likely not responsible for CD, and should not be included in our analysis. We extended a query polypeptide by 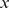 residues (the empirically recommended parameter is 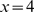, i.e., approximately the period for a helix) on both sides, and investigated whether both a helix and sheet existed in such an enlarged window. Query polypeptides coinciding with this condition were filtered, that is, the corresponding 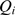 was set to 0. Polypeptides with a high value of 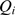 were predicted to be switch sites for structural changes in the query protein corresponding to CD.

The universality of an algorithm is usually proven by analyzing proteins with low sequence identity to those in the learning set. For any input query protein, our algorithm complies with this requirement. In step 1, only segments with sequence identity of less than 26.7% were collected in the RH set. In this way, query data were removed from the prediction process. Moreover, in step 2, samples in set 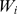 share less than 26.7% sequence identity with those in the RH set. After two approaches to remove redundant data, segments used to calculate the interchange probability were quite different from the query segment. Therefore, the algorithm is an inborn cross validation for any query proteins.

Since the algorithm is based on remote homologues, the region identified should be the same for all members of the corresponding protein family. Thus, there is no strict requirement for the protein structure that needs to be input. The structure of either the protein of interest or its homologues can be used to obtain results for the corresponding protein family.

To ensure a healthy development of modern biology, a patent is applied for corresponding method. We encourage pure scientific research. Contact authors when the method is to be used.

## Supporting Information

Appendix S1 Some details about the algorithm, results and A(H1N1)2009 flu.
(2.73 MB PDF)
