# Supplementary material for: Switch Region for Pathogenic Structural Change in Conformational Disease and Its Prediction
Source: PLoS One. 2010 Jan 11;5(1):e8441. doi: 10.1371/journal.pone.0008441 (PMC2801591; doi:10.1371/journal.pone.0008441)
Supplement: Appendix S1 — Some details about the algorithm, results and A(H1N1)2009 flu. (2.73 MB PDF) [file pone.0008441.s001.pdf]

Supporting information:

1 About the graph of polypeptide relationship, GPR

By sliding a 15-residue window along sequences, proteins were treated as a succession of residue segments. Each 15-residue polypeptide in a representative data set (1612 non-redundant proteins are involved, with sequence identity of less than 25%. The GPR is insensitive to window length, see the paper of donut.) was treated as a node of the graph. An edge was drawn between two nodes if the corresponding polypeptides were remote homologues, and the homologous relationship was identified by a misconnection proof optimization approach. In identifying remote homologs of polypeptides (sequence identity <26.7%), both structural information and homologous similarity derived from knowledge of remote homologous proteins, the well-known BLOSUM30 matrix [1], were considered. We obtained a network of homologous relationships in the whole polypeptide universe, i.e. the GPR.

As the network is composed of 263,577 nodes in total, the GPR is extremely complicated. To give an intuitional picture, a subgraph formed by vital nodes of this network was analyzed topologically in a two-dimensional plane. Homologous polypeptides are close to each other, and non-homologous polypeptides are far apart. The result is shown in the insertion of Figure 1. This subgraph is composed of 10,982 nodes and 169,336 edges in total. According to the approach illustrated in the Methods section, the nodes are classified into nine types and colored differently. There are two nearly separated regions, a helix-donut zone (most members are helix segments and N- and C-terminal helix caps) and a strand-arc zone (mainly comprising $\beta-$sheet segments and N- and C-terminal strand caps). These two regions are sparsely connected by edges emanating from bridge nodes. As nodes directly connect to vertices in the insertion of Figure 1 cover 95.2% of the nodes in the GPR, and the 1612-protein data set is a representative set of all known non-membrane protein structures, features of this subgraph should describe traits of the whole polypeptide universe.

2 About testing set

Clinical reports on actual cases with definite and confirmed nosogenesis are very scarce. References [2, 3, 4, 5, 6] describe a total of 31 proteins responsible for various conformational diseases. Twenty-two of them have usable structural information. Since our method is based on knowledge of non-membrane proteins, this restricts the scope of the application, and four membrane or membrane-associated proteins are unsuitable for our method (amyloid-β precursor protein, α-ketoacid dehydrogenase complex, β-hexosaminidase, α-synuclein). Here we analyzed all other classical proteins that are believed to be responsible for different conformational diseases, and identified regions that cover significant sites for pathogenic structural changes. The conformational diseases can be classified as blood disorders, cardiovascular disease, cerebrovascular disease, neuropathy, encephalopathy, cancer, blindness, and kidney disease, among others.

We treated protein as successive overlapping short residue segments. For each residue segment, we evaluate its ability in arising pathogenic structural change by its probability to jump from the native helix-donut/strand-arc state to the other state. Regions with high interchange probabilities are listed in Table 1, with brief descriptions of their vital roles identified in clinical research. For fibrinogen, we could not find any clinical reports regarding the residues involved in the PDB database. It was difficult to evaluate the predictions for the proteins and hence the result is not listed.

3 Difference between hot spots of fibril formation and switch region

As shown in the example of transthyretin, amyloid-related mutations are not necessarily involved in aggregation-prone regions. It is still not clear whether initial/switch sites occur in hot spots of

aggregation or not. While compared with the small counts of switch sites, aggregation-prone sites are abundant in disease-related proteins. Consequently, according to de Groot et al. [7], at least one-third of residues are involved in predicted hot spots.

4 Detail results

Insulin

Insulin is a peptide hormone with extensive effects on metabolism and many body systems. Insulin injection is used medically to treat some forms of diabetes mellitus. Under solution conditions where the native state is destabilized, this largely helical polypeptide hormone can readily aggregate to form amyloid fibrils with a characteristic cross-β structure. Consequently, it is associated with a clinical syndrome, injection-localized amyloidosis[11].

It was revealed by mass spectrometry analysis that there is a character as insulin forming amyloid fibrils: The disulfide bonds of the native hormone are retained in the amyloid form, providing substantial constraints to refolding. Moreover according to the work of Jimènez et al., a segment donating such disulfide bond constraints plays a significant role in forming the initial aggregates of insulin amyloid fibrils, i.e. is a switch region[12]. As shown in figure S1, it coincides with our prediction that segment 6-20 is the switch region of insulin. Actually this segment is the minimum window that every cysteine responsible for the aforementioned disulfide bond constraints is involved, namely our prediction is very accurate.

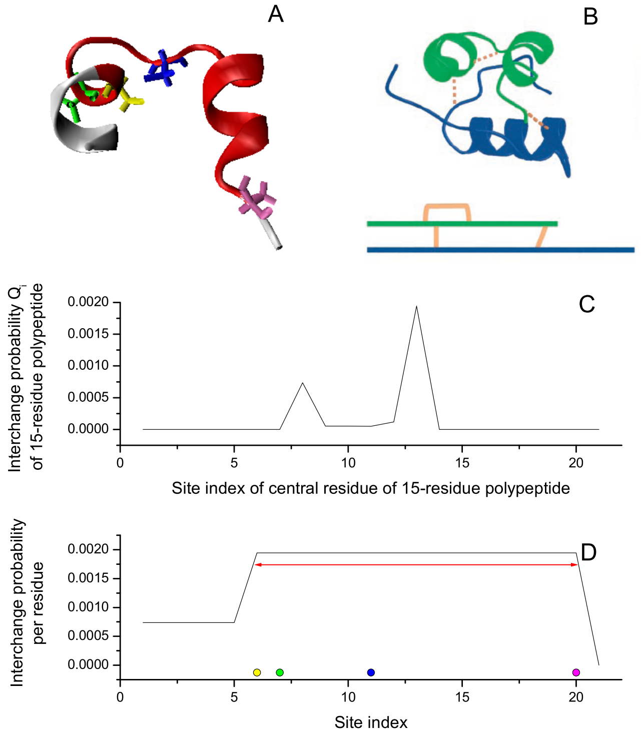

Figure 1: Results of insulin(PDBID: 1ai0_A, 21 residues in length).(A) Structure of insulin. Cysteines responsible for disulfide bonds conserved in refolding are shown in bonds(6yellow, 7green, 11blue and 20magenta). (B) The three native disulfide bonds(gold) shown in insulin structure and topology diagram[12]. (C) Interchange probability for each 15-residue segment indexed by its central residue. (D) The interchange probability for each residue site. In A and D, switch regions predicted are shown in red. This region involves every donor of disulfide bonds which act as constraints to refolding and contribute to initial aggregation of insulin.

Apolipoprotein AI

Apolipoprotein AI(Apo-AI) is the major protein component of high density lipoprotein(HDL) in plasma. The protein promotes cholesterol efflux from tissues to the liver for excretion. As an acceptor for sequential transfers of phospholipids, Apo-AI has two states in vivo(the lipid-free state and the lipid-bound one), with quite different conformations. The lipid-free Apo-AI is comprised of an N-terminal four-helix bundle and two C-terminal helices. Some mutations of Apo-AI can result in hereditary amyloidosis, such as familial amyloid polyneuropathy and familial visceral amyloid[2]. Since the amyloidosis is a consequence of protein aggregation, the lipid-free Apo-AI should be responsible for these diseases. Therefore, it is in the scope that we can cope with.

Stability of lipid-free Apo-AI should be vital for defending amylogenesis of Apo-AI. It is reported that mutations found in human amyloid deposits appear to occur more frequently at the amino terminus of Apo-AI. This is due to the fact that the N-terminal four-helix bundle, especially the helices A:1-43 are essential for the structural stability of lipid-free Apo-AI[14]. Structure of truncated protein △1-43 is quite different from wild type fold. In consequent, there should be some residues in helices A governing the stability of native fold of lipid-free Apo-AI. In helices A, some point mutations at sites 3,10, 13, and 26 result in various clinical consequences[15]. These sites should be in switches in destabilizing native fold of lipid-free Apo-AI. As shown in figure S2, the switch region predicted, #1-15 is an inbuilt segment of helices A. Three out of four aforementioned disease-related sites are in the predicted region. It means our result is correct.

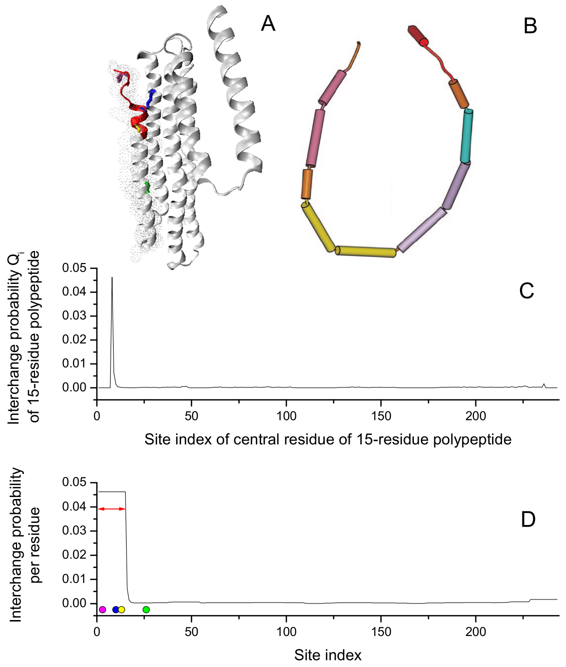

Figure 2: Results of apolipoprotein AI(PDBID: 2a01_A, 243 residues in length).(A) Structure of lipid-free apolipoprotein AI. Helices A:1-43 are vital for structural stability of lipid-free Apo-AI, and are covered by dots. In helices A, disease-related sites are shown in bonds(3magenta, 10blue, 13yellow and 26green). (B) The elliptical loop formed by the △1-43 structure[14]. (C) Interchange probability for each 15-residue segment indexed by its central residue. (D) The interchange probability for each residue site. In A and D, switch regions predicted are shown in red. Three out of four sites responsible for arising disease are in the region we predict.

Calcitonin

Calcitonin is a 32-residue peptide hormone that is being produced by the C-cells of the thyroid and is mainly known for its hypocalcemic effects and the inhibition of bone resorption. Amyloid fibrils of human calcitonin were found to be associated with medullary carcinoma of the thyroid. Calcitonin has little secondary structure at room temperature. However, with a conformational conversion, calcitonin fibrils were found to be highly ordered, consisting of both helix and strand elements.

Recent work indicates a critical role of residue 15-21 for fibril forming and bioactivity of calcitonin[16, 17]. In particular, the conformation and the topological features of side chains of residue 18 and 19 are strongly associated with the self-assembly state, binding affinity and the in vivo hypocalcemic potency of human calcitonin. Another interrelated report is that joint mutations: Y12L, N17H, A26N, I27T, A31T hamper the pathogenic refolding, and result in a non-amyloidogenic analogue of human calcitonin[18]. All the aforementioned sites are important for the disease-related stability of calcitonin. As shown in figure S3, segment 16-31 is identified as switch region in our prediction. This coincide with the aforementioned researches very well.

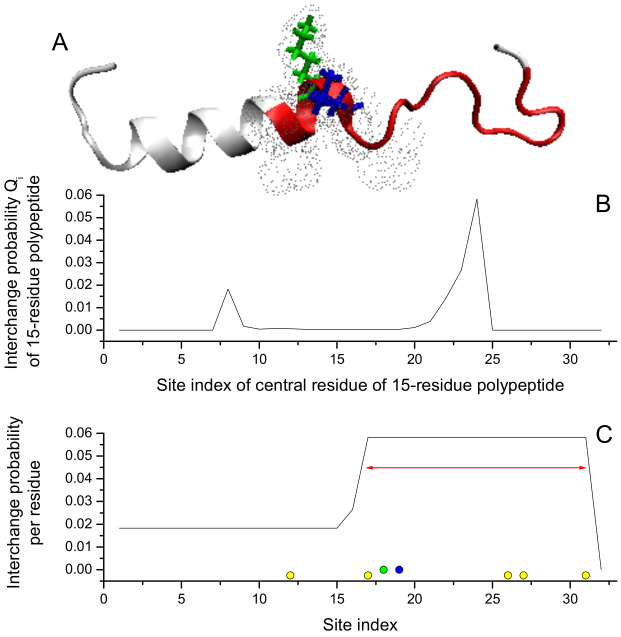

Figure 3: Results of calcitonin(PDBID: 1byv_A, 32 residues in length).(A) Structure of calcitonin. Segment 15-21, shown in dots, governs fibril forming and bio-chemical properties of calcitonin. Wherein, the crucial residues 18 and 19 are shown in bonds, and colored in green and blue respectively. (B)Interchange probability for each 15-residue segment indexed by its central residue. (C) The interchange probability for each residue site. Sites related to the non-amyloidogenic analogue of human calcitonin, namely vital sites in inhibiting the pathogenic refolding are marked in yellow. In A and C, switch regions predicted are shown in red.

Cystatin C

Wild-type human cystatin C is a high-affinity inhibitor of some human cysteine proteases that belong to the papain family, such as cathepsins B, H, K, L, and S. In pathological processes, it forms part of the amyloid deposits in brain arteries that lead to cerebral angiopathy. Patients usually die in their teens from cerebral hemorrhage. The formation of amyloid cystatin C is claimed to be due to conformational changes in the monomer and subsequent domain swapping in the β-fibril structure [19].

According to clinical reports on cystatin C, the most important mutation is at 68 Leu→Gln, which is associated with a severe conformational disease and causes massive amyloidosis, cerebral hemorrhage and death in young adults [20, 21]. Our analysis showed that a peak in interchange probability occurs at window 67 (Figure S4). This means that residues 61–74 around site 68 are critical in this conformational disease, and are related to the initiation of structural changes in view of the double-zone feature of the polypeptide phase space.

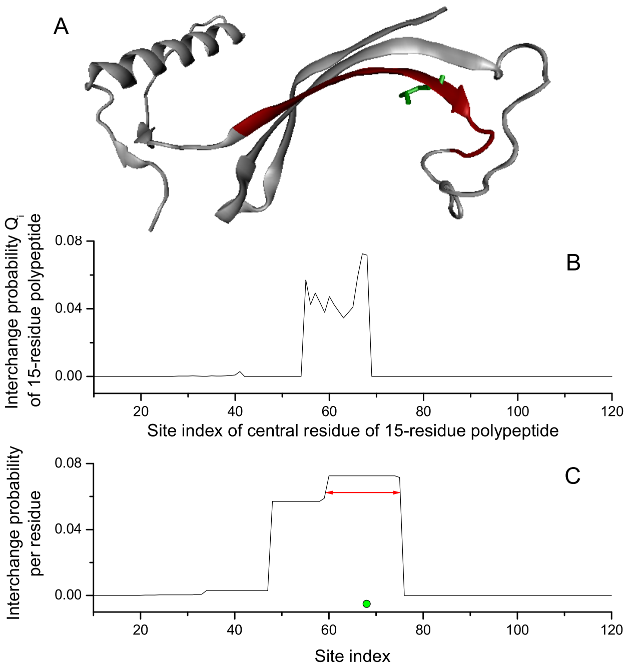

Figure 4: Results of cystatin C(PDBID: 1g96_A, 111 residues in length).(A)Structure of cystatin C. In clinic reports, the most important site is at 68, shown in bonds and green. Mutation L68Q is associated with a severe conformational disease and causes massive amyloidosis, cerebral haemorrhage and death in young adults.(B) Interchange probability for each 15-residue segment indexed by its central residue. (C) The interchange probability for each residue site. In A and C, switch regions predicted are shown in red.

Hemoglobin

Hemolytic anemia is a disorder in which destruction of red blood cells is faster than their production by bone marrow. Many cases of this disease are believed to be due to the presence of unstable hemoglobin that can change its structure and result in disorder. There are various causes of hemoglobin instability, such as single-point mutations, insertion or deletion of amino acids, frame shifts, etc. Unstable hemoglobin molecules lead to varying degrees of hemolysis [22].

Here we analysed the β-chain of human hemoglobin that is believed to be responsible for several hemolytic anemia. The detailed results are shown in Figure S5. Two successive peaks for the interchange probability occur at windows 107 and 118. The two polypeptides overlap and have much higher interchange probabilities (at least two-fold) than the other sites. This means that the switch region for hemoglobin is greater than 15 residues and should be extended. Thus, residues 99–125 were predicted to be prone to conformational changes leading to disorder. This result coincides with clinical reports. According to the database in reference[23], there are totally 43 residue sites for which hemolytic anemia related point mutations have been observed. These sites are interspersed along the 146-residue sequence. In all the corresponding variants, there are four highly unstable mutants(V60E, L110P, A115D, Q127R). Three out of the four are in or close to the region we predicted. It means that the switch region predicted is significant for the structural stability of hemoglobin β-chain.

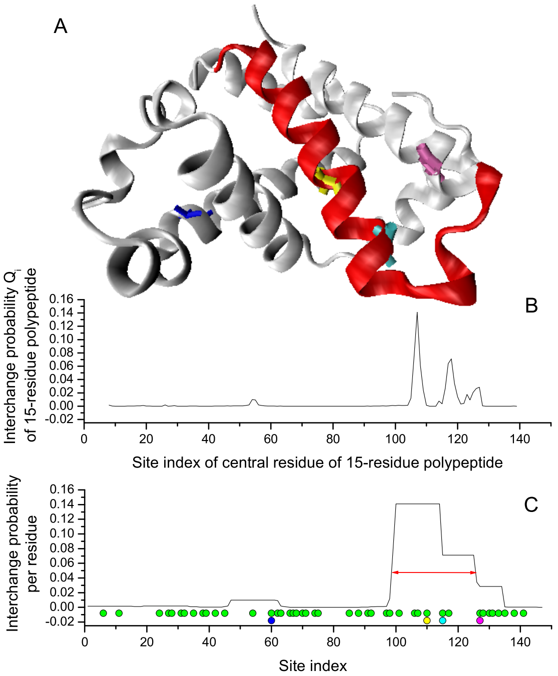

Figure 5: Results of haemoglobin(PDBID: 1xz2_B, 146 residues in length).(A)Structure of haemoglobin β chain. (B) Interchange probability for each 15-residue segment indexed by its central residue. (C) The interchange probability for each residue site. The 43 residue sites for which hemolytic anemia related point mutations have been observed are marked in green. Wherein the highly unstable mutants related sites are shown in various colors(60blue, 110yellow, 115cyan, 127magenta). In A and C, switch regions predicted are shown in red. The highly unstable mutations are prone to occur in or close to the region we predicted. Therefore, disease-related stability of hemoglobin β-chain should be highly sensitive to the region we predicted.

Gelsolin

Gelsolin is an actin-binding protein that is a key regulator of actin filament assembly and disassembly. Wild-type gelsolin is not associated with any amyloid pathology; however, inheritance of some types of mutations, e.g. D187N or D187Y, confers 100% penetrance of Finnish hereditary systemic amyloidosis which is characterized by extensive skin, arterial, neurologic, and ophthalmologic amyloid deposition. Unlike some disease-related proteins which undergo amyloidogenic conformational changes in native fold, gelsolin is associated with amyloidosis due to aberrant cleavage of its precursor protein, i.e. the formation of 70- and 71-residue amyloidogenic gelsolin fragment found in patients. Abnormal cleavage is a direct causation of the disease.

As shown in figure S6, segment 243-264 is predicted to be switch region of gelsolin. It means that disease-related mutation should confer region 243-264 a notable conformational change which produce a sufficient condition for aberrant cleavage, e.g. peptide unique to hydrolase digest. This coincides with the discovery of Page et al. that gelsolin amyloidogenesis is triggered by metalloendoprotease cleavage[24]. The cleavage site is fitly either A^{242}-M^{243} or M^{243}-L^{244}.

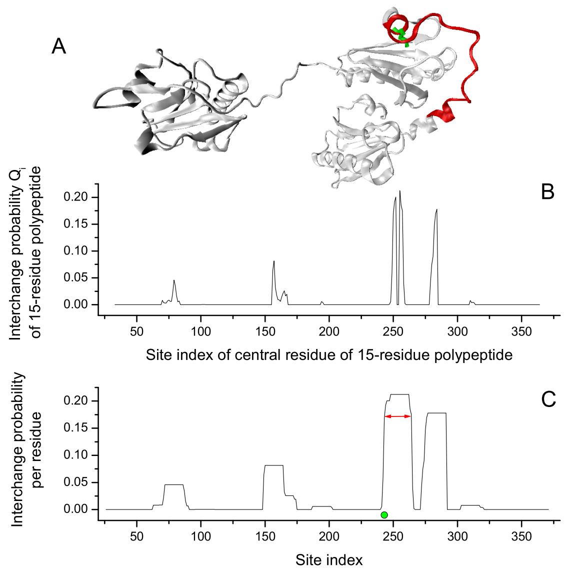

Figure 6: Results of gelsolin(PDBID: 1rgi_A, 346 residues in length).(A)Structure of gelsolin. N-terminus or C-terminus of residue 243 is the cleavage site of gelsolin. The vital residue M243 in triggering amyloidogenesis is shown in bond and colored in green.(B) Interchange probability for each 15-residue segment indexed by its central residue. (C) The interchange probability for each residue site. In A and C, switch regions predicted are shown in red. Triggering site 243 is accurately predicted in gelsolin.

Lysozyme

Lysozyme is an antibacterial protein for which mutations are associated with familial visceral amyloidosis in the liver, spleen, kidneys, and other internal organs. There are five known mutations in the human lysozyme gene that give rise to six variant proteins, 56 Ile→Thr, 57 Phe→Ile, 64 Trp→Arg, 67 Asp→His, 70 Thr→Asn, and the double mutation F57I&T70N. All the variants apart from T70N have been detected in association with amyloid deposits in various human patients.

There are two candidate switch regions, around sites 57 and 67. As shown in figure S7B, compared with structure of wild-type protein, mutation T70N can result in considerable structural rearrangement at sites 68–75 [25] without causing conformational disease. Therefore, the second site is likely not important for the initiation of pathogenic structural changes and can be excluded. In fact, according figure S7B, there is some structural rearrangement at sites 45–51 in the T70N variant, but this is not large enough to cause conformational disease. However the structural rearrangement in this region is very strong for the disease-related mutant D67H, the amyloid donor. This indicates that sites 45–51 correspond to a switch region for lysozyme. As shown in Figure S7, successive peaks for the interchange probability occur at windows 48–50, so that polypeptide 41–57 should be significant in the initiation of structural changes, in agreement with previous research.

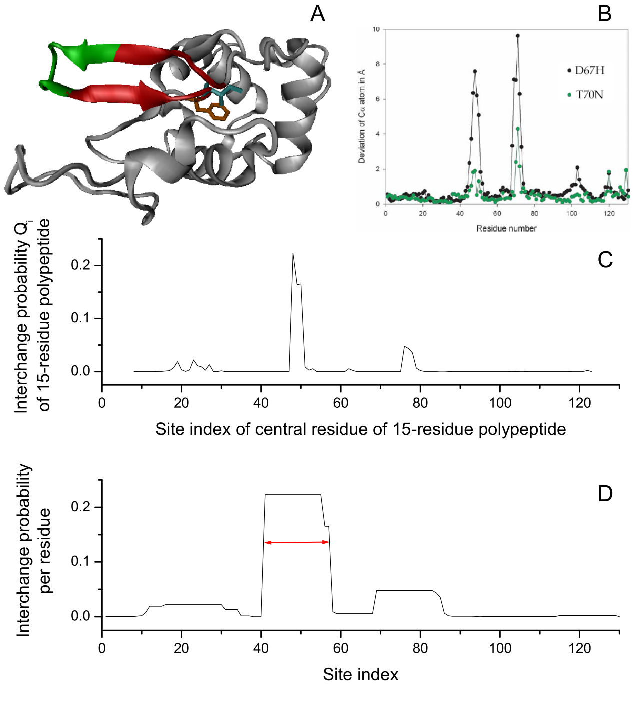

Figure 7: Results of lysozyme(PDBID: 1w08_A, 130 residues in length).(A) Structure of lysozyme. According to Johnson et. al., sites around 45-51, in green should be switch region of lysozyme. (B) A comparison of the crystallographic C^{α} atom deviations for T70N(green,PDB 1W08) and D67H(black,PDB 1LYY) variants, from their positions in the wild-type protein(PDB 1JSF)[25].(C)Interchange probability for each 15-residue segment indexed by its central residue. (D) The interchange probability for each residue site. In A and D, switch regions predicted are shown in red. Disease associated sites(56 cyan and 57 orange) involved in the switch region predicted are also shown in bonds.

Fibrillin-1

Fibrillin-1 is the basic structural element of microfibrils that form a sheath surrounding the amorphous elastin. Mutations in fibrillin-1 is associated with Marfan syndrome that is an autosomal dominant disorder affecting mainly the cardiovascular, skeletal and ocular systems. The range of the clinical severity of Marfan syndrome is strikingly wide. In the most severe form, children of neonatal Marfan syndrome have severe cardiac valve regurgitation and dilation of the proximal aorta,which usually lead to heart failure and death in the first year of life[26].

The structure of the calcium-binding epidermal growth factor-like domains from human fibrillin-1 has been reported in 1996[27]. According to the Marfan database[28], there are three disease-related mutations(D2127E, N2144S, and C2151W) in this domain. As shown in figure S8, the second and third mutates occurs in/near the switch region we predicted.

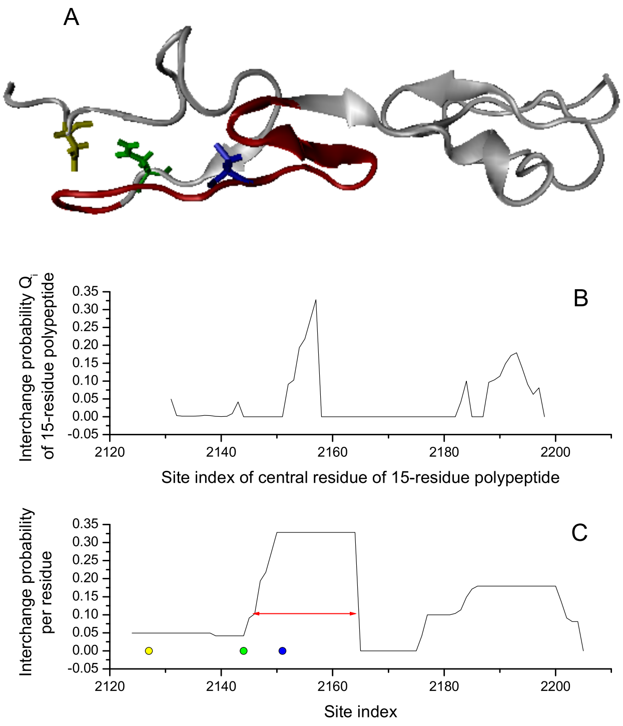

Figure 8: Results of Fibrillin-1(PDBID: 1emn_A, 82 residues in length).(A) Structure of the calcium-binding epidermal growth factor-like domains of filrillin-1. According to Marfan database, the three disease associated sites are shown in bonds. (B)Interchange probability for each 15-residue segment indexed by its central residue. (C) The interchange probability for each residue site. In A and C, switch regions predicted are shown in red. Disease associated sites 2127, 2144 and 2151 are colored in yellow, green and blue respectively.

β_2 microglobulin

β_2 microglobulin is a non-polymorphic light chain of the class I major histocompatibility complex (MHC-I) that plays an important role in the immune system, autoimmunity, and reproductive success [29]. As part of its normal catabolic cycle, β_2 microglobulin dissociates from MHC-I and is transported in serum to the kidneys, where the majority of the protein is degraded. If there is renal failure whereby β_2 microglobulin does not pass through the dialysis membrane, then its clearance from serum is disrupted. This results in an increase in β_2 microglobulin. When a high blood level is maintained for more than 10 years, the protein then self-associates to form amyloid fibrils, causing dialysis-related amyloidosis [30, 31].

It is believed that the formation of amyloid fibrils of β_2 microglobulin accompanies a significant conformational change. There are two successive peaks for the interchange probability at central sites 14 and 16 in Figure S9, indicating that sites 7–23 are likely involved in the initiation of structural changes in β_2 microglobulin. This should correlate with the observation that fragment 21–31 is the well-known amyloidogenic core fragment of β_2 microglobulin [32, 33, 34]. Different from some other proteins, the wild-type β_2 microglobulin can aggregate due to the influence of ageing. In such process, acidification, e.g. in site 17(N→D), is necessary to form amyloid fibrils from both wild type β_2 microglonulin and its variants[35]. It means residues around 17 is significant in triggering pathogenic refolding for both wild-type microglonulin and its variants.

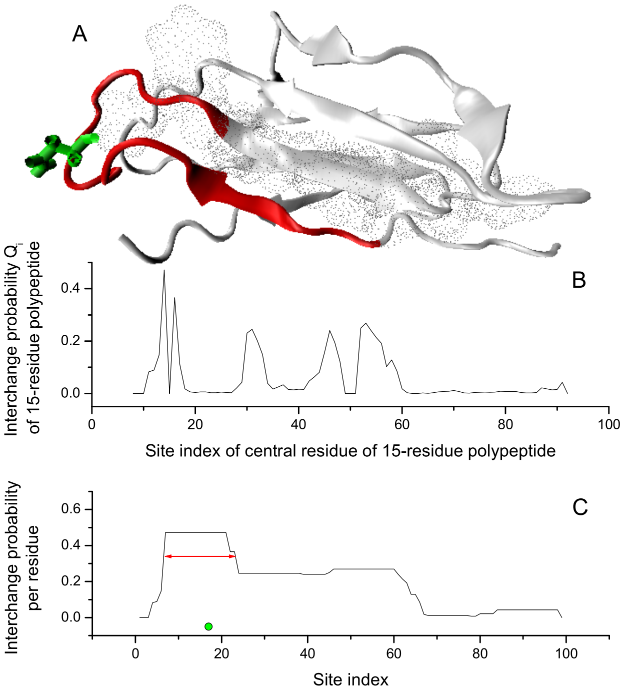

Figure 9: Results of β_2 microglobulin(PDBID: 2vb5_A, 99 residues in length).(A) Structure of β_2 microglobulin. Amyloidogenic core fragment of β_2 microglobulin, 21-31 is shown in dots. The vital residue N17 is shown in bond and colored in green. (B) Interchange probability for each 15-residue segment indexed by its central residue. (C) The interchange probability for each residue site. In A and C, switch regions predicted are shown in red. There are three overlapped residues between the switch region predicted and the amyloid core. Residue N17 is important for the pathological mechanism of amyloid formation of wild-type β_2 microglobulin.

Superoxide dismutase, SOD

The enzyme superoxide dismutase is metalloprotein which catalyzes the dismutation of superoxide into oxygen and hydrogen peroxide. Therefore, it is an important antioxidant defense in nearly all cells exposed to oxygen. There are three major families of superoxide dismutase, depending on the metal cofactor: Cu/Zn type, Fe/Mn type, and Ni type. Some mutations in Cu/Zn SOD enzyme can cause familial amyotrophic lateral sclerosis.

According to reference [36], there are totally 26 residue sites for which disease-related point mutations have ever been reported. As shown in figure S10, segment 37-48 is the region with the highest density of the 26 residue sites. This coincides with our prediction that segment 30-46 should be switch region of pathogenic structural changes for Cu/Zn SOD enzyme.

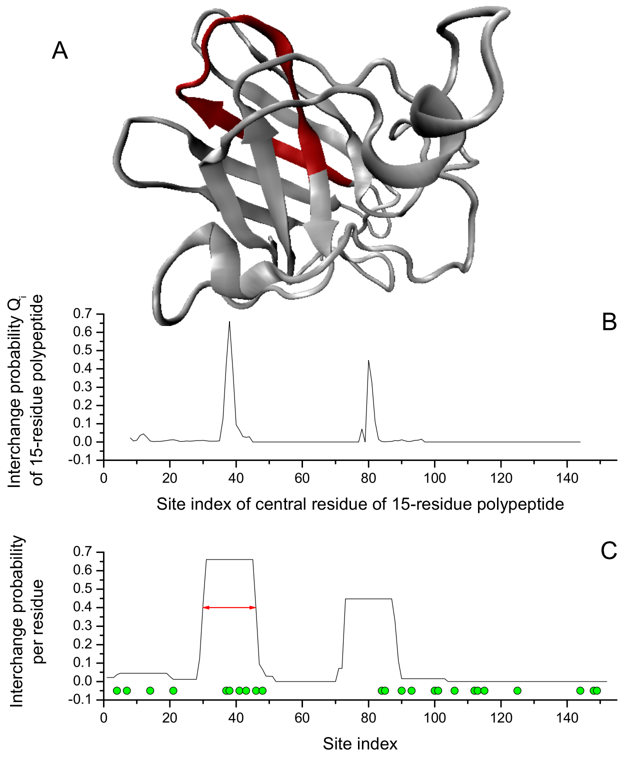

Figure 10: Results of Cu/Zn type superoxide dismutase(PDBID: 2c9v_A, 153 residues in length).(A) Structure of Cu/Zn SOD enzyme. (B) Interchange probability for each 15-residue segment indexed by its central residue. (C) The interchange probability for each residue site. In A and C, switch regions predicted are shown in red. Disease-related sites are marked in C, and in green. The density in segment 37-48 is at least 150% of those of other regions.

Transthyretin

Transthyretin (TTR) is a serum and cerebrospinal fluid carrier of the thyroid hormone thyroxine. It also acts as a carrier of retinol (vitamin A) through an association with retinol binding protein. Amyloid deposition of TTR is associated with several diseases, such as senile systemic amyloidosis, familial amyloid neuropathy, and familial cardiac amyloid [2]. The fibrillar structure resulting from self-association of an abnormal conformation of TTR is thought to be the causative agent in these disorders. The majority of TTR-associated amyloidoses are due to single amino-acid substitutions. In senile systemic amyloidosis, the non-mutated protein is present in amyloid fibrils [37]. However, the mechanism that by which normally soluble TTR tetramers are converted into insoluble amyloid fibrils remains largely unknown.

Here we analyzed normal human TTR to identify sites involved in the initial structural changes in this protein. As shown in Figure S11, polypeptide 46–69 (central residue corresponding to the two successive peaks at sites 53 and 62) was identified as the switch region for conformational changes in TTR. Clinical data demonstrated that the mutation 55 Leu→Pro can cause early-onset familial amyloidotic polyneuropathy [38]. According to clinical reports, L55P is the most notorious mutant, with onset of clinical disease appearing approximately 20 years of age. In comparison, the age of onset is approximately 30 years for V30M carriers and 80 years for wild-type subjects. Analysis based on the crystal structure of the L55P mutant showed that site 55 is important site in the pathway for TTR polymerization to amyloid fibrils [39]. Amyloidogenic regions experimentally determined to date are 10–19 [40] and 105–115 [41], but no segments covering site 55 have been identified so far.

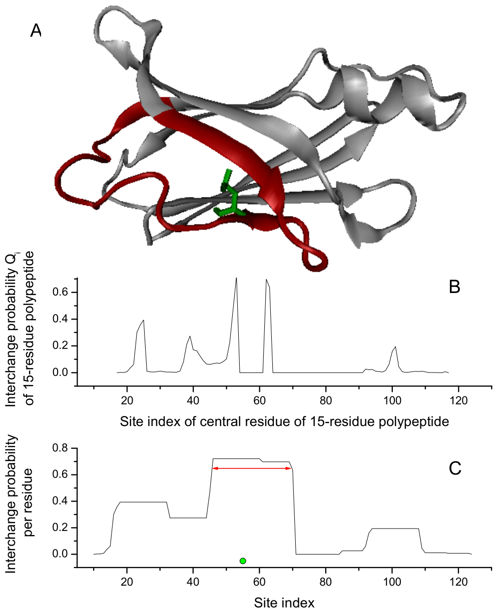

Figure 11: Results of transthyretin(PDBID: 1dvq_A, 115 residues in length).(A) Structure of transthyretin. (B) Interchange probability for each 15-residue segment indexed by its central residue. (C) The interchange probability for each residue site. In A and C, switch regions predicted are shown in red. Site 55 in green is the most important site according to clinic reports. Mutation 55 Leu→Pro is the most notorious mutant, and cause early-onset familial amyloidotic polyneuropathy with onset of clinical disease appearing approximately 20 years of age.

Tumor suppressor protein p53

p53 is a transcription factor in multicellular organisms, where it regulates the cell cycle and thus functions as a tumor suppressor in preventing cancer. As such, p53 has been described as "the guardian of the genome", referring to its role in conserving stability by preventing genome mutation. In about 50% of human cancers, p53 is inactivated as a result of missense mutation in the p53 gene.

Actually, p53 is the most complicated molecule we have ever coped with. As shown in figure S12, there are totaly twenty sites for which the activity of mutants decreases more than 50%[42]. Such sites are interspersed along the 289-residue fold. All mutants without biological activity locate in the N-terminal half(site index > 195) of p53 sequence. Moreover, five of the top six amino acid residues that are most frequently mutated in human cancer are in the N-terminal half(Arg-175, Gly-245,Arg-248, Arg-249, Arg-273,and Arg-282). It means the N-terminal half is significant for conserving the activity of p53(This is largely due to the property conservation of DNA-binding core domain[43]). We predicted region 191-205 to be switch region of p53. The peaks in the N-terminal half are higher than those of the other half. This fits with the aforementioned knowledge qualitatively. Whereas, features in the stability of p53 is extremely complicated. In figure S12, there are several peaks with similar standards of interchange probability. Each of them involves several sites for which the highly destabilizing mutants have been reported[44]. As such, there should be several switch regions in p53, interferefering accuracy of our method by unsuitable basic hypothesis(we suppose there is only one switch region in protein).

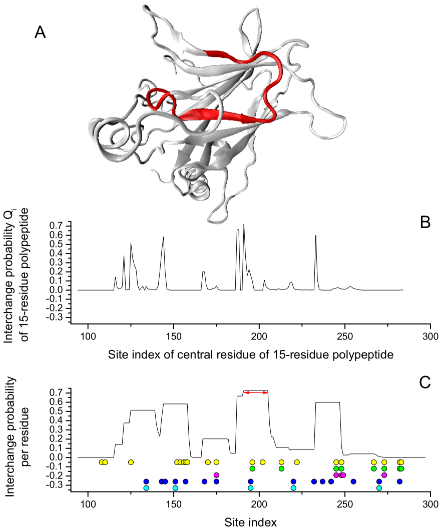

Figure 12: Results of tumor suppressor protein p53(PDBID: 2fej_A, 204 residues in length).(A) Structure of p53. (B) Interchange probability for each 15-residue segment indexed by its central residue. (C) The interchange probability for each residue site. We found p53 is extremely complicated: <**A mass of sites are sensitive to p53's biological activity**> The twenty sites for which the activity of mutants decreases more than half of that of wild-type protein are marked in yellow. Some variants of zero biological activity are produced by point mutation at sites marked in green. <**Nearly every site corresponds to some disease-related point mutations respectively**> Magenta points show sites of the top six most frequently mutated residues in human cancer[42]. <**Various regions are significant for the disease-related stability of p53**> Blue points are sites corresponding to the highly destabilizing mutants reported in[44]. Positions related to the top five most highly destabilizing variants are marked in cyan. In A and C, switch regions predicted are shown in red.

Serpins

Serpins are a family of proteins that inhibit proteases via a profound conformational change that irreversibly locks the protease and serpin together. The normal physiological functions of serpins are based on such highly specific transitions in the natural conformation. Premature conversion of the protein structure will result in a deficiency or dysfunction of the inhibition of proteases, then fibril formation and quite different disease consequences such as emphysema, cirrhosis, and thromboembolic disease [2].

Results of our analysis were shown in Figure S13. Initiation sites for serpins are predicted to be around site 379 (polypeptide 372–386), in agreement with the results of Johnson et al., who reported that wild-type residue Glu-381 plays an important role in stabilising the native, inserted, and activated states of serpin proteins [45]. Actually the predicted region is on the N-terminus of reactive loop of serpins which is vital for the biological properties of the protein.

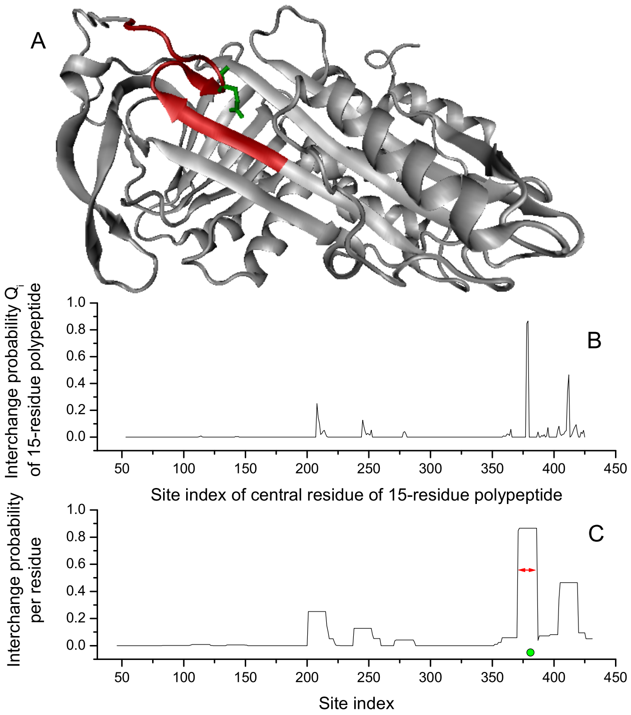

Figure 13: Results of serpins(PDBID: 1e04_A, 386 residues in length).(A) Structure of serpins. (B) Interchange probability for each 15-residue segment indexed by its central residue. (C) The interchange probability for each residue site. In A and C, switch regions predicted are shown in red. Site 381, in green plays an important role in stabilising native, inserted, and activated state of serpins.

Crystallin

Crystallin is a water-soluble structural protein found in the lens of the eye. It is the major protein of the eye lens, accounting for the transparency of lens. Mutations and aging of crystallins cause cataracts, the predominant cause of blindness in the world. In human gamma-D crystallin, there are totally five residue sites for which mutants associated with congenital cataracts have ever reported, i.e. R14, P23, R36, R58, and E106. Three of the five are in the segment 14-36 which we predicted as switch region of gamma-D crystallin(figure S14). Moreover, the threonine substitution in site 23 is reported as the cause of pivotal local conformational and dynamic differences in human gamma-D crystallin[46]. All these evidences prove our result is correct.

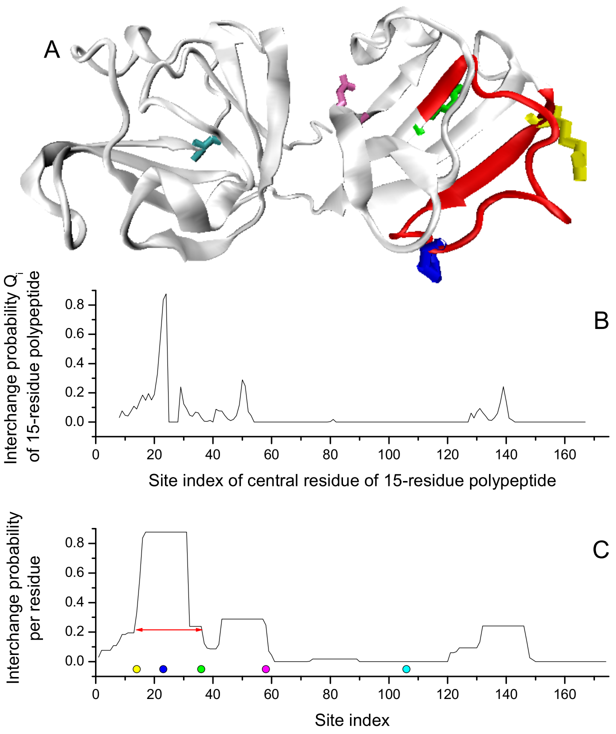

Figure 14: Results of crystallin(PDBID: 1hk0_X, 173 residues in length).(A) Structure of human gamma-D crystallin. The five disease-related sites are shown in bonds(14yellow, 23blue, 36green, 58magenta, and 106cyan). (B) Interchange probability for each 15-residue segment indexed by its central residue. (C) The interchange probability for each residue site. In A and C, switch regions predicted are shown in red.

Low-Density Lipoprotein(LDL) receptor

LDL receptor is a mosaic protein that mediates endocytosis of cholesterol-rich lipoprotein particles. The amino-terminal region of LDL receptor, which consists of seven tandemly repeated cysteine-rich modules(LDL-A modules), mediates binding to lipoproteins. Normally these LDL-A modules extend out into the extracellular fluid, seize the lipoproteins wherein. Then the lipoprotein arrested is imported into the cell by receptor-mediated endocytosis. Mutations of LDL receptor that affect this process result in failure to clear lipoprotein from the circulation, pathologically elevated blood cholesterol and premature heart disease.

Many point mutations that cause familial hypercholesterolaemia map to the fifth LDL-A module of the LDL receptor(LR5). As the module works far from membrane, largely not in a membrane-like environment, we can predict the switch region of LR5 with our method. As shown in figure S15, segment 25-39 is predicted as the switch region of LR5. This coincides with the observation that disease-related point mutations mainly map to a cluster of acidic residues near the carboxy-terminal end of LR5. There are totally eight residue sites for which disease-related point mutations have been observed[13], i.e. illness is induced. Six of them are in the region we predicted.

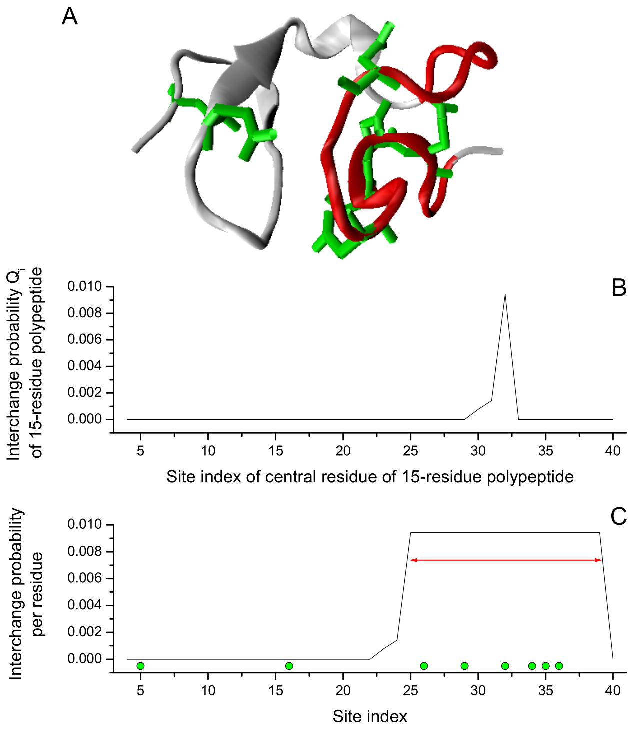

Figure 15: Results of low-density lipoprotein receptor(PDBID: 1ajj_A, 37 residues in length).(A) Structure of low-density lipoprotein receptor. Residue sites related to pathogenic point mutations are shown in bonds, and colored in green. (B)Interchange probability for each 15-residue segment indexed by its central residue. (C) The interchange probability for each residue site. In A and C, switch regions predicted are shown in red. The region we predicted is a segment with highest density of disease-related sites, and should be switch region of low-density lipoprotein receptor.

Cystic fibrosis transmembrane conductance regulator(CFTR)

Cystic fibrosis transmembrane conductance regulator is an ion channel that transports chloride ions across epithelial cell membranes. This transporter-class protein is a complex polytopic membrane protein with several large cytosolic domains, including the regulatory domain R and two cytoplasmically oriented tails, nucleotide binding domains(NBD) 1 and 2. As shown in figure S16B, these domains extend out into the body fluid, and largely in a non-membrane-like environment. Mutations in these domains can disrupt biosynthetic process and result in the cystic fibrosis disease. We have analyzed the NBD1 domain of CFTR. Sites 546-561 were predicted as switch region of NBD1 CFTR. As shown in figure S16D, it is a region with the highest density of missense mutations responsible for cystic fibrosis[47].

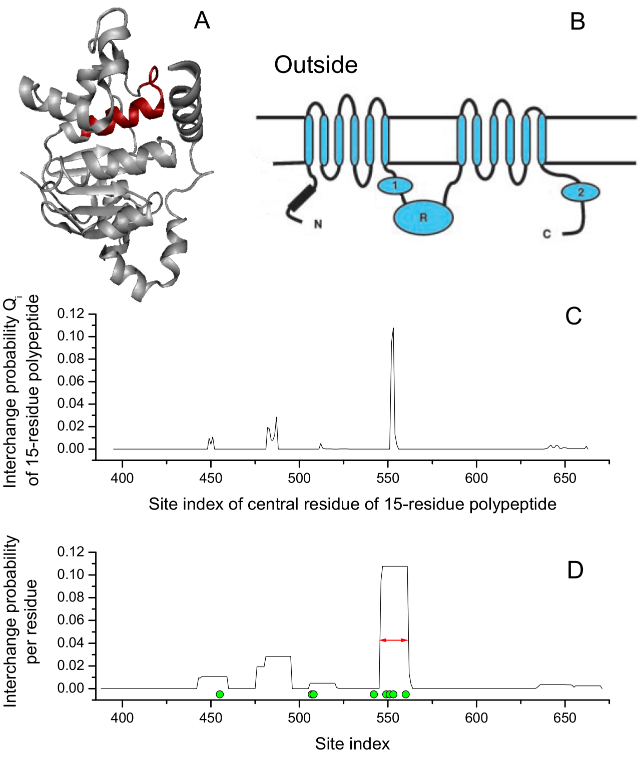

Figure 16: Results of cystic fibrosis transmembrane conductance regulator(PDBID: 1xmi_A, 267 residues in length).(A) Structure of nucleotide binding domain 1 of CFTR. (B) The topology diagram[48] of CFTR. (C) Interchange probability for each 15-residue segment indexed by its central residue. (D) The interchange probability for each residue site. In A and D, switch regions predicted are shown in red. The disease associated sites are shown by green dots.

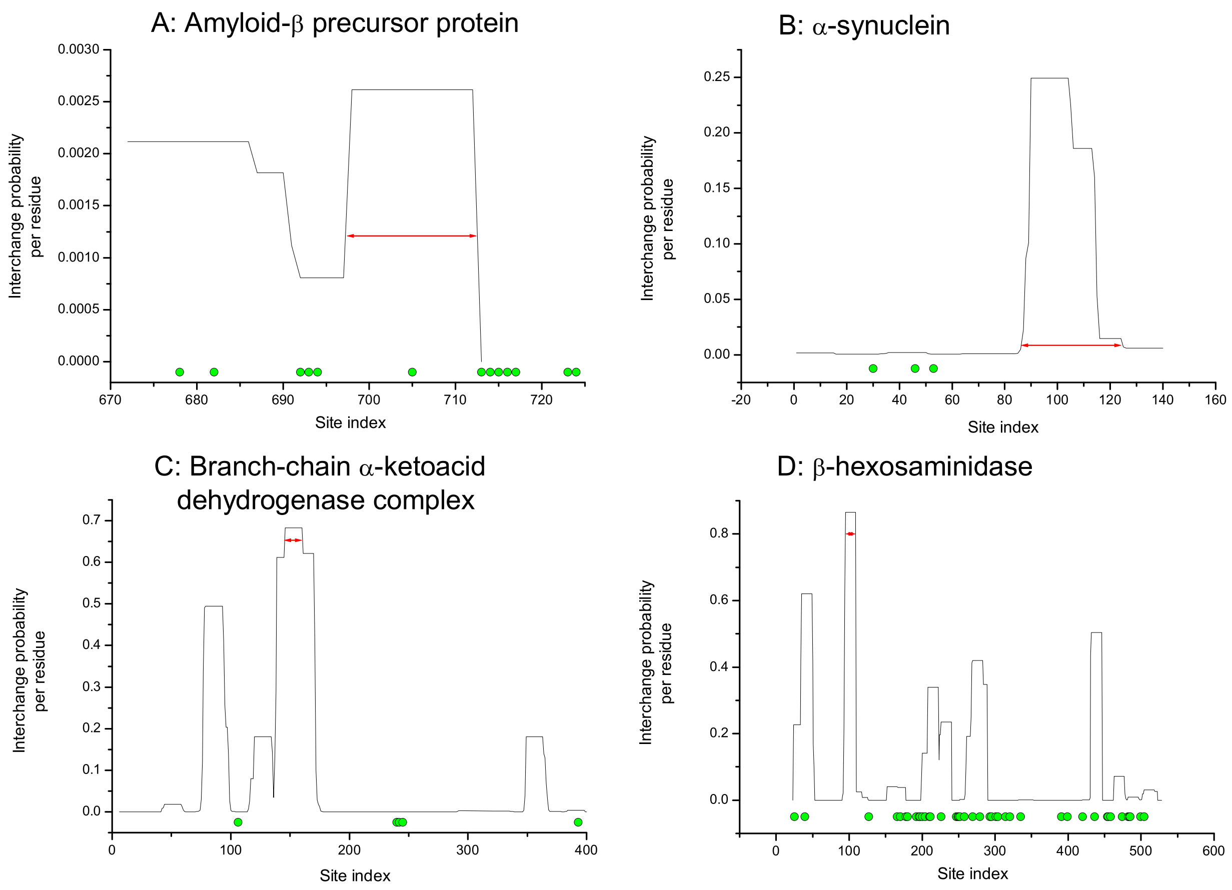

Figure 17: The interchange probability for each residue site of the four unfitting proteins. Switch regions predicted are shown in red. The disease associated sites mentioned in the paper are shown by green dots.

References

[1] Henikoff, S. and Henikoff, J.G. Amino acid substitution matrices from protein blocks. *Proc. Natl. Acad. Sci. USA* **89**, 10915–10919 (1992).

[2] Carrell, R.W. and Lomas D.A. Conformational disease. *Lancet* **350**, 134–138 (1997).

[3] Carrell, R.W., Gooptu, B. Conformational changes and diseases-serpins, prions, and Alzheimer's. *Curr. Opin. Struct. Biol.* **8**, 799–809(1998).

[4] Soto, C. Protein misfolding and disease; protein refolding and therapy. *FEBS lett.***498**, 204–207(2001).

[5] Kelly, J.W. Alternative conformations of amyloidogenic proteins govern their behavior. *Curr. Opin. Struct. Biol.* **6**, 11–17(1996).

[6] Thomas, P.J., Qu, B.H., Pedersen, P.L. Defective protein folding as a basis of human disease. *Trends Biochem. Sci.* **20**, 456–459(1995).

[7] de Groot, N.S., Pallarés, I., Avilés, F.X., Vendrell, J., and Ventura S. Prediction of "hot spots" of aggregation in disease-linked polypeptides. *BMC Struct. Biol.* **5**, 18 (2005).

[8] Liu, X., Zhang, L.M., Guan, S., Zheng, W.M. Distances and classification of amino acids for different protein secondary structures. *Phys. Rev. E.* **67**, 051927 (2003)

[9] Xin, L. and Ya-Pu, Z. Donut-shaped fingerprint in homologous polypeptide relationships - a topological feature related to pathogenic structural conversion of conformational disease. J. Theor. Biol. http://dx.doi.org/10.1016/j.jtbi.2009.02.009

[10] Dobson, C.M. Protein misfolding, evolution and disease. *Trends Biochem. Sci.* **24**, 329–332 (1999).

[11] Dische, F.E., Wernstedt, C., Westermark,G.T., Westermark, P., Pepys, M.B., Rennie, J.A., Gilbey, S.G., Watkins, P.J. Insulin as an amyloid-fibril protein at sites of repeated insulin injections in a diabetic patient. *Diabetologia* **31**, 158–161(1988).

[12] Jiménez, J.L., Nettleton, E.J., Bouchard, M., Robinson, C.V. Dobson, C.M., Saibil, H.R. The protofilament structure of insulin amyloid fibrils. *Proc. Natl. Acad. Sci. USA* **99**, 9196–9201(2002).

[13] Fass, D., Blacklow, S., Kim, P.S., Berger, J.M. Molecular basis of familial hypercholesterolaemia from structure of LDL receptor module. *Nature* **388**, 691–693(1997).

[14] Ajees, A.A., Anantharamaiah, G.M., Mishra, V.K., Hussain, M.M., Murthy, H.M.K. Crystal structure of human apolipoprotein A-I: Insights into its protective effect against cardiovascular diseases. *Proc. Natl. Acad. Sci. USA* **103**, 2126–2131(2006).

[15] Frank, P.G., Marcel, Y.L. Apoliproprotein A-I: structure-function relationship. *J. Lipid Res.* **41**, 853–872(2000).

[16] Haspel, N., Zanuy, D., Ma, B., Wolfson, H., Nussinov, R. A comparative study of amyloid fibril formation by residues 15-19 of the human calcitonin hormone:A single $\beta-$sheet model with a small hydrophobic core. *J. Mol. Biol.* **345**, 1213–1227(2005).

[17] Kazantzis, A., Waldner, M., Taylor, J.W., Kapurniotu, A. Conformationally constrained human calcitonin(hCt) analogues reveal a critical role of sequence 17-21 for the oligomerization state and bioactivity of hCt. *Eur. J. Biochem.* **269**, 780–791(2002).

[18] Andreotti, G., Vitale, R.M., Avidan-Shpalter, C., Amodeo, P., Gazit, E., Motta, A. Designing aggregation-resistant bioactive peptides by three-dimensional structure homology with a non-amyloidogenic analogue. To be Published.

[19] Janowski, R. *et al.* Human cystatin C, an amyloidogenic protein, dimerizes through threedimensional domain swapping. *Nat. Struct. Biol.* **8**, 316–320 (2001).

[20] Abrahamson M. Molecular basis for amyloidosis related to hereditary brain hemorrhage. *Scand. J. Clin. Lab. Invest.* **226**, 47–56 (1996).

[21] Olafsson I., Grubb A. Hereditary cystatin C amyloid angiopathy. *Amyloid Int. J. Exp. Clin. Invest.* **7**, 70–79 (2000).

[22] Outeirino, J., Casey, R., White, J.M., and Lehmann, H. Haemoglobin Madrid beta 115 (G17) alanine–proline: an unstable variant associated with haemolytic anaemia. *Acta. Haematol.* **52**, 53–60 (1974).

[23] http://globin.cse.psu.edu/html/huisman/variants/beta/index.html

[24] Page, L.J., Suk, J.Y., Huff, M.E., Lim, H.J., Venable, J., III, J.Y., Kelly, J.W., Balch, W.E. Metalloendoprotease cleavage triggers gelsolin amyloidogenesis. *EMBO J.* **24**, 4124–4132(2005).

[25] Johnson R.J.K. *et al.* Rationalising lysozyme amyloidosis: insights from the structure and solution dynamics of T70N lysozyme. *J. Mol. Biol.* **352**, 823–836 (2005).

[26] Putnam, E.A., Cho, M., Zinn, A.B., Towbin, J.A. Byers, P.H. Milewicz, D.M. Delineation of the Marfan phenotype associated with mutations in exons 23-32 of the FBN1 gene. *Am. J. Med. Genet.* **62**, 233-242 (1996).

[27] Downing, A.K., Knott, V., Werner, J.M., Cardy, C.M., Campbell, I.D., Handford, P.A. Solution structure of a pair of calcium-binding epidermal growth factor-like domains: implications for the Marfan syndrome and other genetic disorders. *Cell* **85**, 597-605 (1996).

[28] Collod-Béroud, G. *et al.* Marfan Database (third edition): new mutations and new routines for the software. *Nucleic Acids Res.* **26**, 229–233 (1998).

[29] Bjorkman, P.J. *et al.* Structure of the human class I histocompatibility antigen, HLA-A2. *Nature* **329**, 506–512 (1987).

[30] Floege, J., and Ketteler, M. β2-Microglobulin-derived amyloidosis: An update. *Kidney Int.* **59**, 164–171 (2001).

[31] Gejyo, F., et al. A new form of amyloid protein associated with chronic hemodialysis was identified as beta 2-microglobulin. *Biochem. Biophys. Res. Commun.* **129**, 701–706 (1985).

[32] Hasegawa, K. *et al.* Amyloidogenic synthetic peptides of β2-microglobulina role of the disulfide bond. *Biochem. Biophys. Res. Commun.* **304**, 101–106 (2003).

[33] Hiramatsu, H., Goto, Y., Naiki, H., and Kitagawa, T. Core structure of amyloid fibril proposed from IR-microscope linear dichroism. *J. Am. Chem. Soc.* **126**, 3008–3009 (2004).

[34] Hiramatsu, H., Goto, Y., Naiki, H., and Kitagawa, T. Structural model of the amyloid fibril formed by β_2-microglobulin #21-31 fragment based on vibrational spectroscopy. *J. Am. Chem. Soc.* **127**, 7988–7989 (2005).

[35] Kad, N.M., Thomson, N.H., Smith, D.P., Smith, D.A. , Radford, S.E. β_2- microglobulin and its deamidated variant, N17D form amyloid fibrils with a range of morphologies in vitro. *J. Mol. Biol.* **313**, 559–571(2001).

[36] de Belleroche, J., Orrell, R., King, A. Familial amyotrophic lateral sclerosis/motor neurone disease (FALS): a review of current developments. *J. Med. Genet.* **32**, 841–847(1995).

[37] Saraiva, M.J.M. Transthyretin mutations in health and disease. *Hum. Mutat.* **5**, 191–196 (1995).

[38] Jacobson, D.R., McFarlin, D.E., Kane, I., and Buxbaum, J.N. Transthyretin Pro55, a variant associated with early-onset,aggressive, diffuse amyloidosis with cardiac and neurologic involvement. *Hum. Genet.* **89**, 353–356 (1992).

[39] Sebastiao, M.P., Saraiva, M.J., Damas, A.M. The crystal structure of amyloidogenic Leu55 $\rightarrow$ Pro transthyretin variant reveals a possible pathway for transthyretin polymerization into amyloid fibrils. *J. Biol. Chem.* **273**, 24715–24722 (1998).

[40] Jarvis, J.A., Kirkpatrick, A. and Craik, D.J. 1H NMR analysis of fibril-forming peptide fragments of transthyretin. *Int. J. Pept. Protein Res.* **44**, 388–398 (1994).

[41] Jaroniec, C.P., MacPhee, C.E., Astrof, N.S., Dobson, C.M. and Griffin, R.G. Molecular conformation of a peptide fragment of transthyretin in an amyloid fibril. *Proc. Natl. Acad. Sci. USA* **99**, 16748–16753 (2002).

[42] http://p53.free.fr/Database/p53_database.html

[43] Joerger, A.C., Ang, H.C., Fersht, A.R. Structural basis for understanding oncogenic p53 mutations and designing rescue drugs. *Proc. Natl. Acad. Sci. USA* **103**, 15056–15061(2006).

[44] http://www-p53.iarc.fr/stability.html

[45] Johnson, D.J.D., and Huntington, J.A. The influence of hinge region residue Glu-381 on antithrombin allostery and metastability. *J. Biol. Chem.* **279**, 4913–4921 (2004).

[46] Jung, J., Byeon, I.J., Wang Y., King, J., Gronenborn, A. The structure of the cataract causing P23T mutant of HgD crystallin exhibits local distinctive conformational and dynamic changes. *Biochemistry* DOI: 10.1021/bi802292q.

[47] http://www.genet.sickkids.on.ca/cftr/app

[48] Cormet-Boyaka, E., Jablonsky, M., Naren, A.P., Jackson, P.L., Muccio, D.D., Kirk, K.L. Rescuing cystic fibrosis transmembrane conductance regulator (CFTR)-processing mutants by transcomplementation. *Proc. Natl. Acad. Sci. USA* **101**, 8221-8226 (2004).

5 Protein selection for 2009 A(H1N1)

Besides NP, four inner proteins are also reported to be important for host specificity: M1, PA, PB1, and PB2.

The matrix protein M1 is one of the proteins encoded by influenza A virus RNA segment 7 and highly conserved. Only 5% of the 252-amino acid-long M1 proteins differ among the divergent flu virus strains. M1 protein interacts with viral RNP and surface membrane glycoproteins. M1 was found to be involved in the regulation of RNP nuclear-cytoplasm transporting, virus assembly and budding[1, 2]. M1 is the major targets of influenza-specific CTL recognition in human and its amino acid substitutions have been found to be associated with escape from human CTL[3, 4].

The influenza A viral polymerases which function as heterotrimeric complex formed by PA, PB1 and PB2 play a crucial role in viral transcription and replication. The interactions of the complex with host factors implied their involvement in host specificity [5, 6, 7].

We also predicted switch regions of the four proteins: M1 PDBID 1EA3_A, PA PDBID 2ZNL_A, PB1 PDBID 2ZNL_B, and PB2 PDBID 3CW4_A, with identities 95%, 96%, 93% and 95% to those proteins of A/California/04/2009(H1N1) respectively. But the residue segments in A/California/04/2009(H1N1) corresponding to their switch regions have been expressed by virus of human host ever. So, according to our algorithm, they should not be responsible for the host alteration with a similar reason as that of S1 NP 2009 A(H1N1). Therefore only NP is analyzed in detail.

References

[1] Helenius A. Unpacking the incoming influenza virus.Cell. 1992;69(4):577-8.

[2] Ye ZP, Baylor NW, Wagner RR. Transcription-inhibition and RNA-binding domains of influenza A virus matrix protein mapped with anti-idiotypic antibodies and synthetic peptides. J Virol. 1989;63(9):3586-94

[3] Gotch, F., Rothbard, J., Howland, K., Townsend, A., McMichael, A. Cytotoxic T lymphocytes recognize a fragment of influenza virus matrix protein in association with HLA-A2. Nature.1987;326:881-2.

[4] Berkhoff EG, de Wit E, Geelhoed-Mieras MM, Boon AC, Symons J, Fouchier RA, Osterhaus AD, Rimmelzwaan GF. Functional constraints of influenza A virus epitopes limit escape from cytotoxic T lymphocytes. J Virol. 2005;79(17):11239-46.

[5] Gabriel G, Herwig A, Klenk HD. Interaction of polymerase subunit PB2 and NP with importin alpha1 is a determinant of host range of influenza A virus. PLoS Pathog. 2008 ;4(2):e11.

[6] Honda A, Okamoto T, Ishihama A. Host factor Ebp1: selective inhibitor of influenza virus transcriptase. Genes Cells. 2007;12(2):133-42.

[7] Naito T, Momose F, Kawaguchi A, Nagata K.Involvement of Hsp90 in assembly and nuclear import of influenza virus RNA polymerase subunits. J Virol. 2007;81(3):1339-49.

Table S1. All nonredundant human strains with single point mutants of S2 of virus 2009 A(H1N1) selected from the top 5000 strains in NCBI according to E-value of BLAST. Some redundant strains similar to these viruses are deleted.

Segment corresponding to S2	Strains	Host	Other information	Isolation time	Isolation place
TRGVQIASNENVETM	A/California/04/2009(H1N1)		present		
TRGVQIASNENVEaM	A/New Jersey/8/1976(H1N1)	Most: pigs Some: human and birds	Caused an abortive epidemic in humans in USA in 1976	1957-2000	Global
	A/Wisconsin/3523/1988(H1N1)	Human	From human vaccines	1980s	North American
	A/Maryland/12/1991(H1N1)	Human	From human vaccines	1990s	North American
	A/Wisconsin/10/98 (H1N1)	Sporadic human	Triple reassortant among human,avian, and swine virus	1998	North American
	A/Iowa/CEID23/2005(H1N1)	Major: pigs Minor:Human	Triple reassortant among human,avian, and swine virus	2005	North American
	A/Hong Kong/1073/99(H9N2)	Major: birds Sporadic human	avian virus	1999	South China
	A/Hong Kong/156/97(H5N1)	Major: birds Sporadic human	avian virus	1997-2009	Southeast Asia
TRGVQIASNENmETM	A/Brevig Mission/1/1918(H1N1)	Major: pigs and human pandemic	avian virus, cause 1918 Spanish flu	1918	Global
	A/Wilson-Smith/1933(H1N1)	Human	The first flu virus isolated in human	1933	Global
	A/Puerto Rico/8/34(H1N1)	Human	Some viruses were isolated in the 1980s and 1990s.	Major: 1930s	Global
	A/Melbourne/35(H1N1) A/Henry/1936(H1N1)	Human	All genes is closely related to A/Puerto Rico/8/34(H1N1)	Major: 1930s	Global
	A/Bel/1942(H1N1)	Human	An exception was isolated in 1990s in England from a pig.	1942-1985	Global
	A/Iowa/1943(H1N1)	Human	All genes is closely related to A/Puerto Rico/8/34(H1N1)	1943-1985	Global
	A/Mongolia/231/85(H1N1) A/Mongolia/153/88(H1N1)	Human	A reassortant between A/Puerto Rico/8/34(H1N1) and A/USSR/77(H1N1) isolated in 1977 pandemic	1980-1988	Global
	A/Mongolia/111/91(H1N1)	Human	All genes is closely related to A/Puerto Rico/8/34(H1N1)	1991	Mongolia
	A/Switzerland/8808/2002(H1N1)	Sporadic human	Swine virus	2002	Switzerland
	A/Thailand/271/2005(H1N1)	Major: pigs Minor:Human	A reassortant between North American and Eurasian swine virus	2005	Eurasian
	A/Philippines/344/2004(H1N2)	Sporadic Human	Further reassortant between reassortant H3N2 and classical H1N1 swine virus	2004	Eurasian
	A/Victoria/1968(H3N2)	Human	Cause 1968 flu pandemic	1968-2009	Global
	A/Hong Kong/1774/99(H3N2)	Sporadic Human	Triple reassortant among human influenza virus, a Eurasian lineage avian virus.	1999	Eurasian
	A/human/Zhejiang/16/2006(H5N1)	Major: birds Sporadic human	Avian virus	1997-2009	Southeast Asia
